# Tailoring the optical and dynamic properties of iminothioindoxyl photoswitches through acidochromism†

**DOI:** 10.1039/d0sc07000a

**Published:** 2021-02-09

**Authors:** Miroslav Medved', Mark W. H. Hoorens, Mariangela Di Donato, Adèle D. Laurent, Jiayun Fan, Maria Taddei, Michiel Hilbers, Ben L. Feringa, Wybren Jan Buma, Wiktor Szymanski

**Affiliations:** University Medical Center Groningen, Department of Radiology, Medical Imaging Center, University of Groningen Hanzeplein 1 9713 GZ Groningen The Netherlands w.szymanski@umcg.nl; Centre for Systems Chemistry, Stratingh Institute for Chemistry, Faculty of Science and Engineering, University of Groningen Nijenborgh 7 9747 AG Groningen The Netherlands; Regional Centre of Advanced Technologies and Materials, Faculty of Science, Palacký University in Olomouc Šlechtitelů 27 CZ-771 46 Olomouc Czech Republic miroslav.medved@umb.sk; Department of Chemistry, Faculty of Natural Sciences, Matej Bel University Tajovského 40 SK-97400 Banská Bystrica Slovak Republic; Laboratoire CEISAM UMR UN-CNRS 6230, Université de Nantes Nantes F-44000 France; European Laboratory for Non Linear Spectroscopy (LENS) via N. Carrara 1 50019 Sesto Fiorentino Italy; ICCOM-CNR *via* Madonna del Piano 10 50019 Sesto Fiorentino (FI) Italy; Van't Hoff Institute for Molecular Sciences, University of Amsterdam Science Park 904 1098 XH Amsterdam The Netherlands; Radboud University, Institute for Molecules and Materials, FELIX Laboratory Toernooiveld 7c 6525 ED Nijmegen The Netherlands

## Abstract

Multi-responsive functional molecules are key for obtaining user-defined control of the properties and functions of chemical and biological systems. In this respect, pH-responsive photochromes, whose switching can be directed with light and acid–base equilibria, have emerged as highly attractive molecular units. The challenge in their design comes from the need to accommodate application-defined boundary conditions for both light- and protonation-responsivity. Here we combine time-resolved spectroscopic studies, on time scales ranging from femtoseconds to seconds, with density functional theory (DFT) calculations to elucidate and apply the acidochromism of a recently designed iminothioindoxyl (ITI) photoswitch. We show that protonation of the thermally stable *Z* isomer leads to a strong batochromically-shifted absorption band, allowing for fast isomerization to the metastable *E* isomer with light in the 500–600 nm region. Theoretical studies of the reaction mechanism reveal the crucial role of the acid–base equilibrium which controls the populations of the protonated and neutral forms of the *E* isomer. Since the former is thermally stable, while the latter re-isomerizes on a millisecond time scale, we are able to modulate the half-life of ITIs over three orders of magnitude by shifting this equilibrium. Finally, stable bidirectional switching of protonated ITI with green and red light is demonstrated with a half-life in the range of tens of seconds. Altogether, we designed a new type of multi-responsive molecular switch in which protonation red-shifts the activation wavelength by over 100 nm and enables efficient tuning of the half-life in the millisecond–second range.

## Abstract 2

Protonation of an ITI molecular photoswitch shifts its absorption to the orange/red part of the spectrum and allows for manipulation of the thermal half-life of the photogenerated *E*-isomer over three orders of magnitude.

## Graphical abstract

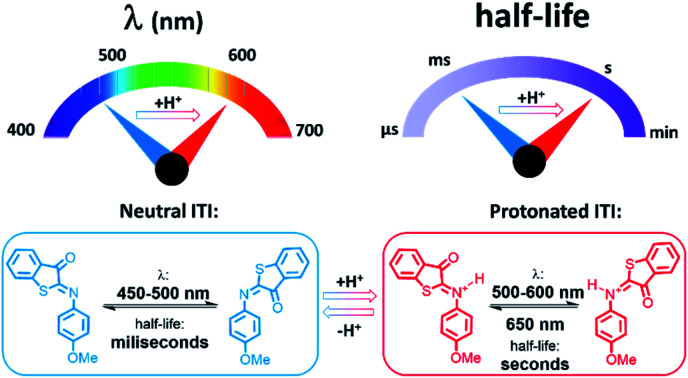

## Introduction

Due to the outstanding spatiotemporal resolution offered by light delivery, molecular photoswitches^1,2^ are widely used to precisely control the function of molecules and systems in chemistry,^3,4^ biology^5–7^ and materials science.^8,9^ Yet, an even higher level of molecular control can be obtained by combining light with a second external stimulus such as the binding of a proton to the molecular photoswitch. Protonation of molecular photoswitches has important consequences for their spectral properties, isomerization mechanism and half-life of the meta-stable form, and has been recognized as a simple and reversible method for tuning the photochromes for particular applications. These efforts are often inspired by nature, where protonation plays a key role in the switching function of *e.g.* retinal Schiff bases,^10^ fluorescent proteins,^11^ proton pumps,^12^ and phytochromes.^13^

**Fig. 1:**
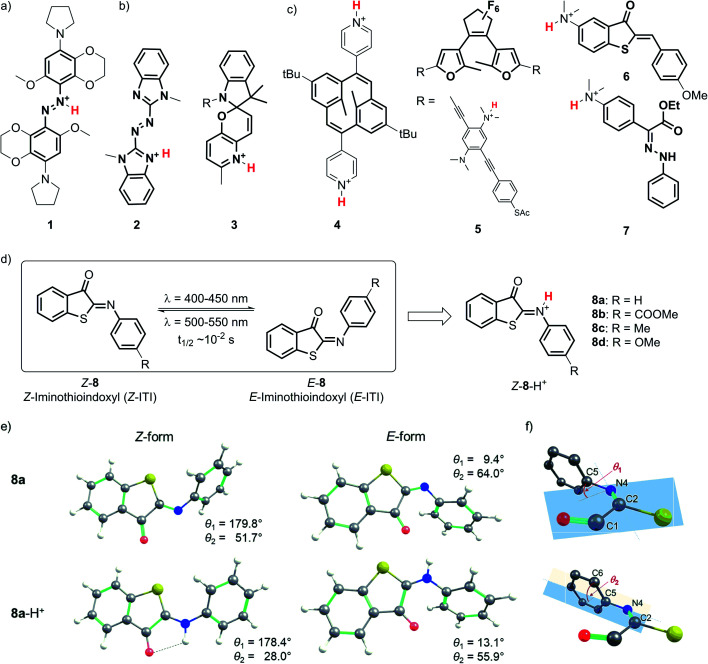
Structures of protonated molecular photoswitches, representative of azobenzene (**1**), azoheteroarene (**2**), spiropyran (**3**), dihydropyrane (**4**), diarylethene (**5**), hemithioindigo (**6**) and hydrazone (**7**) types. Structures are shown in which protonation occurs at the isomerizing bond (a), switch core (b), and substituents (c). (d) Switching of the iminothioindoxyl switch **8** between the thermally stable *Z* form and the metastable *E* form, and the structure of its protonated form *Z*-**8**-H^+^. (e) Structures of the *Z* and *E* forms of **8a** and **8a**-H^+^ in DCM optimized at the M06-2X/6-31+G(d)/SMD level of theory. (f) The numbering of atoms and definitions of dihedral angles *θ*_1_ and *θ*_2_.

Various types of molecular photoswitches (Fig. 1) have been shown to functionally respond to protonation, which can occur at the isomerizing bond (Fig. 1a), at the photoswitch core (Fig. 1b) or at the substituents (Fig. 1c). The first case is exemplified by *tetra-ortho*-alkoxy-azobenzene **1** (Fig. 1a) introduced by Woolley and co-workers,^14,15^ the protonation of which has enabled its operation with near-infrared light. Inducing a bathochromic shift has also been achieved by the transfer of a proton to the core of the chromophore in azoheteroarene **2** ^16^ and to the substituent in dihydropyrane **4** (Fig. 1).^17^ In the latter case, protonation also improved the photoswitching quantum yield, which was observed in the case of oligo(phenylene ethynylene) (OPE)-embedded diarylethene **5** as well.^18^

From an application perspective, the relation between protonation and photoswitching can be exploited in both directions: protonation can be used to modulate the switching process and thereby create multi-responsive systems,^19^ while photoisomerization can be employed to change the proton affinity of a molecule in reversible photoacids and bases.^20^ Examples of the first approach include hemithioindigo **6**,^21^ in which protonation of the strongly electron-donating dimethylamino group changes its electronic character, thus inducing a band shift and turning **6** from a P-type into a T-type switch; similar effects were observed for hydrazone **7**.^22^ The photoacid designs are most often based on modified spiropyrans^23^ such as compound **3**,^24^ while photoswitchable basicity has been realized with azobenzenes^25^ and azoheteroarenes.^26^

Here we explore the influence of protonation on iminothioindoxyl (ITI), a new member of the family of visible-light-operated photoswitches.^27^ The ITI photoswitch consists of half a thioindigo and half an azobenzene (Fig. 1d), which results in a photo-isomerizable C=N double bond. This fused photoswitch features both the visible light absorption of thioindigo and the band separation characteristics of azobenzene, resulting in a fully-visible-light photoswitch with a 100 nm band separation between the *Z* and *E* isomers. The *Z* isomer can be switched to the *E* isomer using light of around 400–450 nm while back-isomerization can be done photochemically (typically in the 500–550 nm range) or thermally, with a half-life of tens of milliseconds at room temperature. The small size of ITI would enable its future introduction into functional systems without affecting their overall properties. However, the fast re-isomerization process constitutes a challenge for many applications, as it limits the concentration of the metastable *E* isomer that can be achieved under irradiation. For example, based on MRI and PET data of mean transit times for blood in the cerebral vasculature,^28^ Woolley and co-workers suggested^15^ that the optimal thermal half-life should be in the 0.1–10 s range to provide an optimal spatial resolution. This is an order of magnitude higher than that found for ITI. Furthermore, bathochromically shifting the absorption maximum of ITI would allow switching with non-destructive, low-energy visible/red/NIR light and thus greatly expand the scope of applications. Moreover, it would enable selective ITI operation in complex mixtures with other components absorbing in the UV-violet-blue, and thus offer unique possibilities for the design of orthogonal switching systems.^29^

Detailed understanding of the isomerization mechanism enables the tuning of photoswitches through molecular engineering and pathway selection. In the context of ITI, the fast *E*–*Z* re-isomerization is assumed^27^ to proceed through thermal in-plane inversion of the imine nitrogen atom.^30–32^ In this process, a linear intermediate is formed, after which the imine relaxes to the thermally favored *Z* state. With this in mind, we hypothesized that engaging the imine-nitrogen electron pair in a transient covalent bond with a proton would inhibit fast re-isomerization and enhance the thermal stability of the metastable *E* isomer. Furthermore, inspired by previous reports,^15,16^ we also expected it to result in a significant bathochromic shift of the absorption bands of both isomers, thereby meeting our second objective.

In this work, we present a combined experimental and theoretical study of the photochemistry of protonated ITI switches. Using steady-state and time-resolved spectroscopies on time scales ranging from femtoseconds to seconds in combination with time-dependent density functional theory (TD-DFT) calculations, we unravel the mechanistic and spectral aspects of ITI acidochromism and showcase ITI variants that feature optimal half-lives of several seconds and absorption spectra extending into the 600–700 nm region.

## Results and discussion

### Protonation of the nitrogen atom of ITI results in a bright S_1_ absorption band that extends to >550 nm

**Fig. 2:**
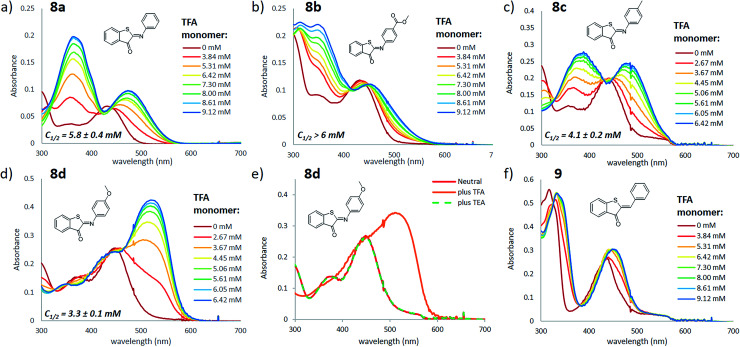
Titration of ITI photoswitches **8a–d** (panels a–e) and hemithioindigo **9** (panel f) with TFA in DCM (20 μM). The legend of each graph describes the concentration of TFA monomers based on the association constant of 1.5 l mol^−1^ reported in ref. 34 (see Section S2 of the ESI† for further details). The spectra with total TFA concentration are presented in Section S3 of the ESI.† Reversible protonation of compound **8d** (panel e) was achieved by addition of TFA (total concentration of 200 μM) to 20 μM ITI, followed by addition of TEA (excess). The *C*_1/2_ values represent the concentration of the TFA monomer at which half of the ITI molecule is protonated, obtained from fitting the titration curve with a sigmoid curve (see Section S3 of the ESI† for further details), with standard deviation values representing the uncertainty of fitting.

To investigate the influence of protonation on the spectral properties of ITI switches, we titrated a range of compounds **8a–d** (Fig. 1) with trifluoroacetic acid (TFA) in dichloromethane (Fig. 2). The chosen compounds included unsubstituted ITI (**8a**) and ITI derivatives containing either electron-withdrawing (EWG, **8b**) or electron-donating (EDG, **8c,d**) groups in the *para* position of the phenyl ring. Importantly, as discussed further in Section S2 of the ESI,† the titration curves in Fig. 2 are plotted against the calculated concentration of the TFA monomer, which is more representative of the solution acidity than the total TFA concentration due to the self-association of TFA in DCM.^33,34^

Neutral ITIs **8a–d** feature two absorption bands in their UV spectra: a strong one at *λ* ∼400–450 nm associated with the S_0_ → S_2_ electronic transition, and a weak one at *λ* ∼350 nm which is attributed to the S_0_ → S_3_ transition (*vide infra*).^27^ Upon addition of TFA to the ITI solutions, we observed the emergence of two new absorption bands: one at roughly the same spectral region as the S_0_ → S_3_ transition in the neutral form, and one that is significantly red-shifted and is observed in the 450–550 nm region, tailing to over 600 nm in the case of methoxy-substituted ITI **8d**. Intriguingly, the relative intensities of the emerging bands strongly depend on the electronic nature of the substituent at the phenyl ring. For the EWG-substituted system **8b**, the higher energy band at *λ* ∼350 nm dominates the spectrum, but this effect is smaller for unsubstituted ITI **8a** and further decreases with the increasing electron donating character of the substituents. For compound **8c**, featuring a weakly electron-donating methyl substituent, the bands are almost equal in intensity. The even stronger electron-donating character of the methoxy group in compound **8d** results in a pronounced absorbance of the red-shifted band, while in the UV region of the spectrum no increase is observed.

In a qualitative manner (Fig. 2), the proton affinity of ITIs follows the electronic properties of the studied compounds as can be observed by comparing the concentration of the TFA monomer needed to protonate half of the ITI population (*C*_1/2_, Fig. 2). Such a comparison shows that the increase of the electron density on the nitrogen atom in the order of ITIs **8b**–**8a**–**8c**–**8d** is accompanied by an increased proton affinity. Furthermore, the effects observed upon titration with TFA can be fully reversed by addition of triethylamine (TEA) (Fig. 2e). These observations demonstrate that the effects observed by UV-vis spectroscopy indeed originate from protonation of ITI molecules **8**.

Aiming to further exclude the influence of non-specific solvatochromic effects, and to confirm the role of the nitrogen atom as the protonation site in ITI switches, we used the homologous hemithioindigo switch **9** as a model compound that does not feature the C=N double bond. Addition of TFA to a solution of compound **9** in DCM (Fig. 2f) resulted in only minor spectral changes that did not feature the emergence of two bands observed for compounds **8**. To gain more insight into the observed effects, we have evaluated the influence of TFA addition to compounds **8a** and **9** by NMR spectroscopy (see Section S4 of the ESI†). Acidification of a solution of **8a** in CD_2_Cl_2_ resulted in the emergence of a new set of NMR signals, indicative of the formation of another species. Conversely, when TFA was added to a solution of **9**, no new signals emerged and only a down-field shift of the initial signals could be observed, in line with the solvatochromic effect. Altogether, these observations confirm the protonation of compounds **8** at the nitrogen atom, which leads to strongly pronounced changes in the UV-vis spectra. Importantly, we observed that addition of an acid leads to the appearance of a bathochromically shifted absorption band that extends into the red-light region of the electromagnetic spectrum (*λ* > 600 nm) as was previously observed for azobenzene compounds.^14,15^

### Irradiation of the protonated *Z*-ITI results in the isomerization to the *E* form

**Fig. 3:**
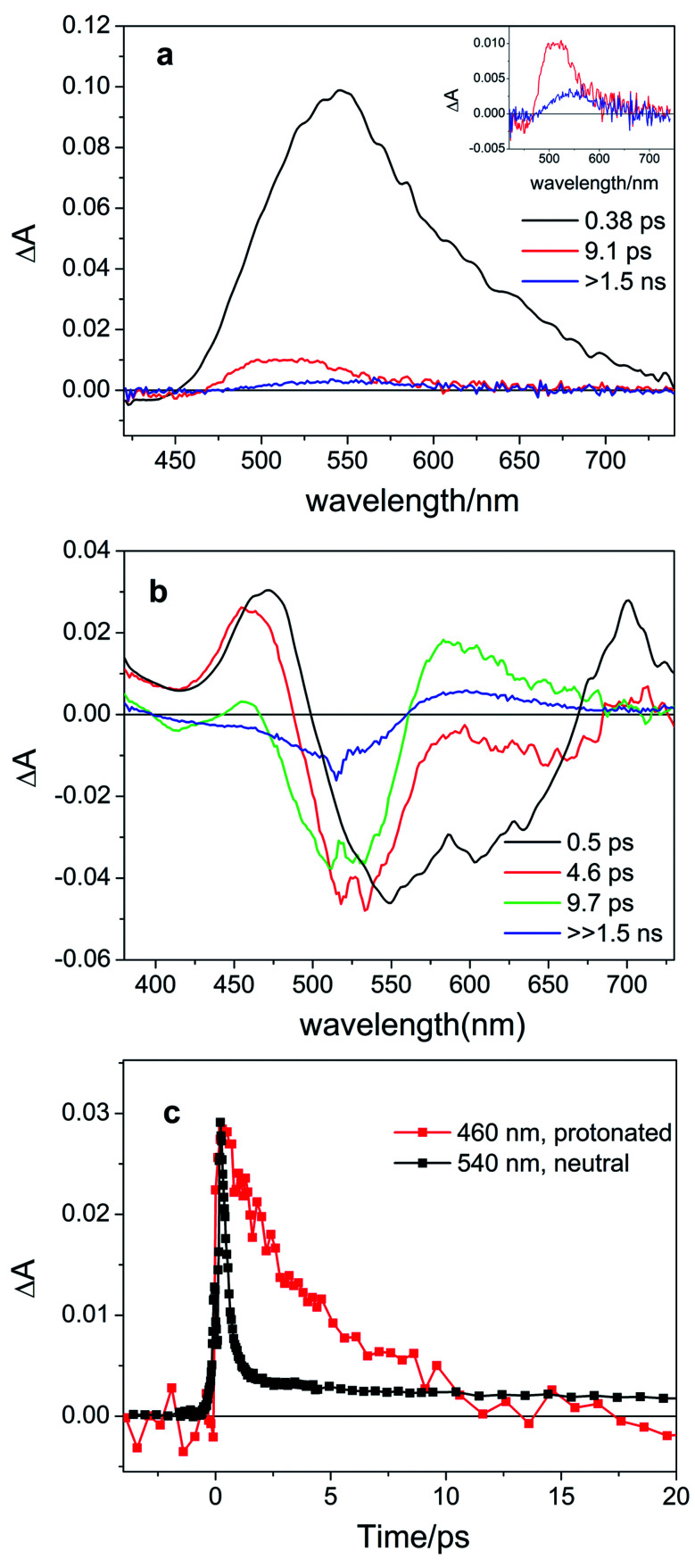
Evolution Associated Difference Spectra (EADS) obtained from global analysis of the ultrafast transient absorption data recorded for (a) neutral compound **8d** in DCM excited at 400 nm (the long-living component is enhanced in the inset) and (b) protonated compound **8d**-H^+^ (0.64 M TFA in DCM, corresponding to the 14.4 mM TFA monomer^34^) excited at 514 nm. Panel (c) shows the comparison of kinetic traces recorded at the maximum of the excited state absorption band for neutral (black line) and protonated (red line) compound **8d**.

Next, we investigated the effects of light irradiation on the spectra of protonated compounds using transient absorption spectroscopy with sub-ps time resolution. The Evolution Associated Difference Spectra (EADS) obtained from global analysis of the data recorded for protonated compound **8d**, which featured the most pronounced absorbance of the red-shifted band, are shown in Fig. 3, while those of other compounds (**8a**, **8c**) are shown in the ESI (Fig. S5.1 and S5.2†).

For the previously investigated neutral compounds, transient absorption spectroscopy indicated that isomerization is an ultrafast process, occurring with a mechanism very similar to that described in the case of azobenzene.^27^ The time-resolved spectra of the neutral compounds, as shown in Fig. 3a for compound **8d**, are dominated by a very intense broad excited-state absorption band (ESA), which, according to the global analysis of the recorded kinetic traces, decays on a very fast 0.38 ps timescale. In line with our previous interpretation, we assume that for neutral samples excitation with visible light populates the bright S_2_ excited state, which undergoes internal conversion to S_1_ on an ultrafast timescale. Isomerization then proceeds on a time scale competing with vibrational relaxation in S_1_. The molecule thus relaxes to the ground state of either the *Z* or *E* isomer where vibrational cooling takes place in about 9 ps. The long-living spectral component displays a differential line-shape because of the emergence of a red-shifted positive product band assigned to the *E* isomer^27^ (see Fig. 3a, inset). Compounds **8a** and **8c** have a similar behavior, see Fig. S5.4 in the ESI.†

**Fig. 4:**
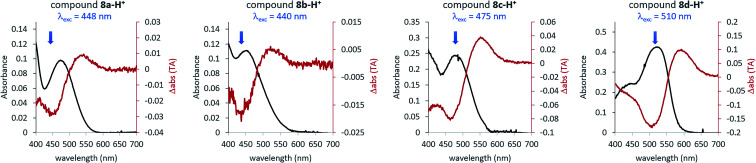
Overlay of the UV-vis spectra of protonated ITI compounds **8** (black lines) (0.155 M TFA in DCM, corresponding to the 7.0 mM TFA monomer^34^) and the results of ns-TA measurements (red lines) obtained upon excitation with light at the wavelength indicated with the blue arrow.

According to our calculations (*vide infra*), for protonated compounds, the excitation with visible light populates the S_1_ state, which gains oscillator strength upon protonation. Importantly, ultrafast transient absorption spectra show also for protonated compound **8d** the emergence of a positive product band (Fig. 3b green and blue lines) upon relaxation of the excited state absorption band. The emergence of this band, which retains the large (>60 nm) separation from the parent band typical for the neutral ITIs (blue EADS in Fig. 3a), is characteristic for the formation of the metastable *E* isomer and leads to a differential signal on the nanosecond timescale whose negative part is a band located at the absorption position of the stable *Z* isomer, indicative of the drop of its population.^27^ On a longer timescale, the formed differential signal is in line with what is observed in ns-transient absorption measurements (Fig. 4, *vide infra*), showing that the observed spectral changes and band separation are consistent throughout the studied range of compounds.

The isomerization kinetics for protonated ITIs appears to be quite different compared to their neutral counterparts, as exemplified in Fig. 3b for compound **8d** and as evidenced by the comparison of kinetic traces recorded at the excited state absorption band of the neutral and protonated compound, reported in Fig. 3c. Directly after excitation, the transient spectra of compound **8d**-H^+^ are characterized by a positive excited state absorption (ESA) band peaking at about 460 nm and a negative broad band assigned to the convolution of bleaching and stimulated emission. On a fast 0.5 ps timescale, the stimulated emission mostly recovers, while the ESA slightly blue shifts as a result of a fast relaxation process that brings the system out of the Frank–Condon region. During the subsequent evolution, occurring on the 4.6 ps timescale, a positive band peaking at 580 nm emerges, while the ESA band mostly decays. We associate this evolution with the reaching of a conical intersection (CI) from which the molecule relaxes to the *Z* and *E* ground states. In the ground state, the system undergoes vibrational relaxation within 9.7 ps. As mentioned above, the long-living spectral component is interpreted in terms of a differential *E*–*Z* ground state absorption. The nature of the substituent on the phenyl ring has also an influence on the excited state decay kinetics, but we do not observe a clear distinction among EWG and EDG moieties. Indeed, our measurements show that the slowest kinetics is measured in the case of the unsubstituted compound **8a**-H^+^, for which the ESA band recovers in about 40 ps. Faster dynamics is observed for compound **8c**-H^+^ and a further acceleration is noticed for compound **8d**-H^+^ (ESI Fig. S5.5†). Shifting the excitation wavelength to 400 nm has instead only a minimal influence on the excited state dynamics (ESI Fig. S5.1–3†).

To gain deeper insight into the effects of protonation on the geometrical features and the nature of electronic transitions in compounds **8a–d**, we employed density functional theory (DFT) for the investigation of the key stationary points on the ground state (GS) potential energy surface (PES), and its time-dependent counterpart (TD-DFT) for the analysis of the vertical excitations and excited state (ES) geometry optimizations (see Section S1.2 for Computational details†). All (TD-)DFT calculations were performed using the Gaussian09 ^35^ and Gaussian16 ^36^ programs. The calculations employing wavefunction-based methods were carried out using the Orca program (ver. 4.2.1)^37,38^ and Turbomole^39^ (ver. 7.3).

The optimized GS structures and the relative energies of the *Z* and *E* forms revealed remarkable variations between protonated and neutral compounds **8**. In general, the protonated forms are less distorted than their neutral counterparts (Fig. 1e, S8.1 and S8.2†). The differences are more pronounced in the *Z* form, where the dihedral angle *θ*_2_ (defined in Fig. 1f) is in the range of 21–28° and 37–60° for *Z*-**8**-H^+^ and *Z*-**8**, respectively (Table S2†). Such a change is attributed to the efficient delocalization of the positive charge in the protonated *Z* species owing to the larger spreading of the π-electron density from the phenyl group in the less distorted arrangement (a negative mesomeric effect, Fig. S8.3 and Table S8.2†). This effect is less pronounced in the *E* form due to the steric hindrance between a hydrogen atom on the phenyl ring and the oxygen atom on the carbonyl group. The stability of *Z*-**8**-H^+^ is further enhanced by the presence of a weak hydrogen bond between the N–H hydrogen and C=O oxygen atom (Fig. 1e). Consequently, the relative stability of the *Z* form with respect to the *E* form of **8**-H^+^ is by 2–3 kcal mol^−1^ higher than in the case of their neutral analogs (Table S8.1†).

**Fig. 5:**
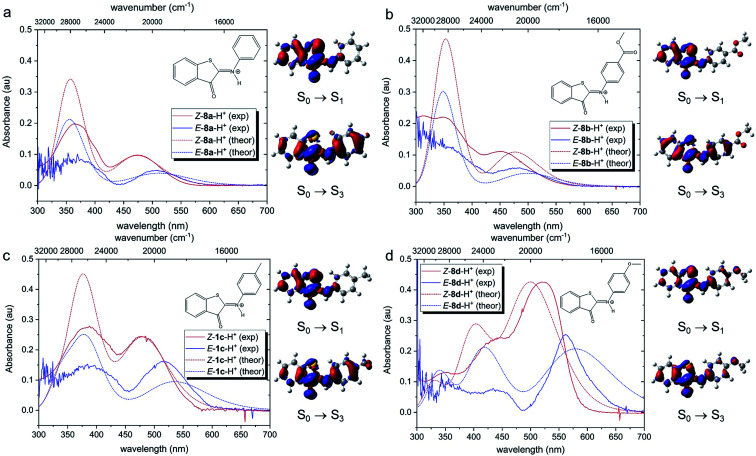
Comparison of experimental and theoretical electronic absorption spectra of **8**-H^+^ in DCM. The theoretical spectra were obtained at the TD-M062X/6-311++G(2df,2p)/SMD//M06-2X/6-31+G(d)/SMD level of theory (FWHM = 4000 cm^−1^). All simulated spectra were red-shifted by 2800 cm^−1^, so that the S_0_ → S_1_ vertical excitation energy of *Z*-**8a**-H^+^ coincided with the experimental *λ*_max_ value of the first (long-wavelength) band. For each compound, they were normalized to the absorption intensity of the first band of the *Z* isomer, *i.e.*, the same scaling factor was used for the corresponding *E* isomer. The experimental spectra of the *E* forms have been obtained by subtracting the absorption spectra of the *Z* forms from the respective *E*–*Z* transient absorption spectra. Electron density difference (EDD) plots for the three lowest transitions are also displayed (red = decrease, blue = increase, isovalue = 0.0015 au).

Theoretical analysis of the electronic transitions performed at the TD-M06-2X/6-311++G(2df,2p)/SMD level^40–42^ provides a consistent rationalization of the impact of protonation of ITIs on their absorption spectra. As reported earlier,^27^ in the case of neutral *Z*-**8** compounds, the band observed at *λ* ∼400–450 nm corresponds to the S_0_ → S_2_ transition with prevailing π → π* (HOMO → LUMO) character (Table S8.8†). The first excited state S_1_, which is dark in neutral **8a** and **8b** (for **8c** and **8d** it acquires intensity owing to a smaller structural distortion), is a mixed state with a significant n → π* contribution, where n represents to a large extent the lone pair on the nitrogen atom (Fig. S8.12–S8.19†). As this lone pair is protonated in the acidic environment, the n(*N*) → π* transition is no longer possible in protonated *Z*-**8**-H^+^. As a result, the S_1_ state with predominantly ππ* character becomes bright (Fig. 5, see also Tables S8.5, S8.7 and Fig. S8.4–S8.11†) and the corresponding transition is therefore red-shifted compared to the S_0_ → S_2_ transition in neutral ITIs (Table S8.6†), as observed in Fig. 2. The S_2_ state in *Z*-**8**-H^+^ is dark owing to its nπ* character, but the S_3_ state (ππ*) is again bright (Fig. 5, Table S8.7†) and manifests itself as the second band observed experimentally (Fig. 2). It is gratifying to note that the calculated shifts upon protonation and their dependence on the nature of the substituent are in excellent agreement with our experimental observations. Also, the intensity ratio of the two bands in the *Z*-**8(a–d)**-H^+^ series is well reproduced by the calculations (Fig. 5) and can be attributed to a different ππ* or nπ* and charge transfer (CT) character of the two transitions (see Section S8.4 in the ESI†).

Fig. 5 shows that also for the protonated *E*-**8**-H^+^ forms the theoretically predicted spectra are in good agreement with experimental observations, providing further support for the adopted theoretical approach. As in the case of *Z*-**8**-H^+^, the two observed bands for *E*-**8**-H^+^ can be attributed to S_0_ → S_1_ and S_0_ → S_3_ transitions. However, in *E*-**8**-H^+^, the S_1_ state has a mixed ππ*/nπ* character owing to a more twisted structure (*cf.* Table S8.2†) and the oscillator strength of the S_0_ → S_1_ transition is therefore significantly smaller compared to *Z*-**8**-H^+^. Comparison of the S_0_ → S_1_ absorption maxima shows that for *E*-**8**-H^+^ these bands are red-shifted with respect to the *Z*-**8**-H^+^ form, in particular for EDG derivatives (see Table S8.5 and further comments in Section S8.4 in the ESI†).

In the next step, we addressed possible pathways of *Z*-**8a**-H^+^ after S_0_ → S_1_ excitation. Geometry optimization of the S_1_ state led to a minimum that is structurally very similar to that of the GS (Fig. S8.20†). Importantly, the N–H bond length did not appear to be affected by the excitation (Fig. S19a†), suggesting similar acidity of the molecule *before* and *after* the excitation, and thus precluding deprotonation in the excited state. Nevertheless, the electronic density difference (EDD) plot for the S_0_ → S_1_ transition (Fig. 5a) indicated an increased electron density on the carbonyl oxygen, so an excited-state intramolecular proton transfer (ESIPT) process could not be excluded. A relaxed scan along the O⋯H coordinate revealed an energetic barrier of *ca.* 19 kcal mol^−1^, after which the system proceeded to the CI region in the ESIPT geometry (Fig. S8.21†). Such an energetic barrier is significantly higher than that for torsion along the dihedral angle *θ*_1_, which was estimated to be *ca.* 3 kcal mol^−1^ (Fig. S8.22†). Therefore, we conclude that direct photo-isomerization through a CI appears to be much more plausible than a pathway involving ESIPT.

### Protonation of *E*-ITI extends its half-life through blocking the nitrogen inversion

**Fig. 6:**
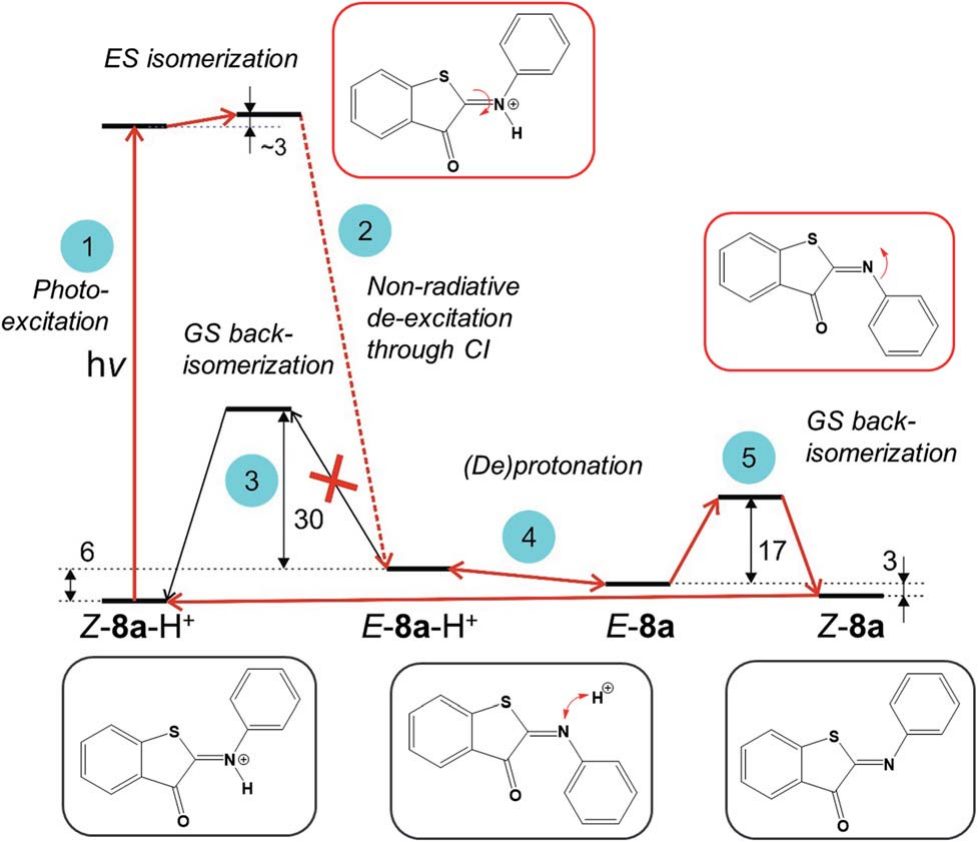
Reaction mechanism of photoisomerization of ITIs in an acidic environment as revealed by theoretical investigations. The GS energy values (in kcal mol^−1^) reported here for compound **8a** in DCM were obtained employing the composite DLPNO-CCSD(T)/M06-2X/SMD approach. See the text for computational details and discussion of individual steps.

Next, we evaluated if engaging the nitrogen electron pair in a bond with the proton is a viable strategy to increase the thermal stability of the metastable *E*-ITI isomer. Based on theoretical investigations we propose a model including the photo-activation step as well as the GS kinetics and (de)protonation equilibria as displayed in Fig. 6 that is consistent with all experimental observations.

The analysis of the S_1_ PES for *Z*-**8a**-H^+^, which excluded ESIPT as well as ES deprotonation, implies that photo-activation of the protonated *Z* form (step 1) leads – through a CI – to the formation of an *E*-**8**-H^+^ isomer (step 2), which can either thermally back-isomerize (step 3) or deprotonate (step 4). The activation barrier for back-isomerization of *E*-**8a**-H^+^ to *Z*-**8a**-H^+^ determined by the composite DLPNO-CCSD(T)/M06-2X/SMD approach^43–45^ is *ca.* 30 kcal mol^−1^, discarding this pathway for *E*-**8a**-H^+^ as well as for the other derivatives (Table S8.1†). The transition state exhibits a pyramidal structure (Fig. S8.1†) and not a linear arrangement as in the neutral species. Interestingly, however, the calculated Δp*K*_a_ values of *Z*-**8**-H^+^ and *E*-**8**-H^+^ suggest that the *E* form is more acidic compared to *Z*, and that this difference is more pronounced for EDG derivatives (Table S8.3†). It can therefore be expected that the acid–base equilibrium between *E*-**8**-H^+^ and *E*-**8** (step 4) is readily established, opening an alternative path for the back-isomerization in the deprotonated form (step 5). Indeed, the activation barrier for this step (nitrogen inversion) is about 17 kcal mol^−1^ for **8a** (Table S8.1†), *i.e.*, significantly lower than that for the back-isomerization of the protonated form. After the formation of *Z*-**8a**, the cycle is closed by protonation of *Z*-**8a** back to *Z*-**8**-H^+^. An important consequence of this mechanism is that it predicts that the half-life of the *E* isomer can be tuned by adjusting the proton concentration. By increasing the acidity of the environment, the acid–base equilibrium (step 4) is shifted to the left, *i.e.*, towards the protonated *E*-**8**-H^+^ form, thus decreasing the population of *E*-**8** available for back-isomerization.

**Fig. 7:**
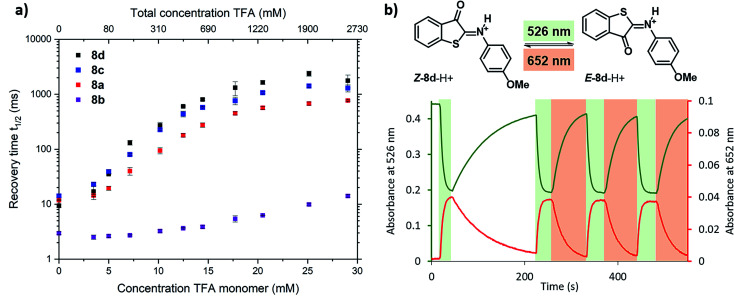
Photoswitching behaviour of ITIs **8a–d**. (a) Dependence of the recovery half-life time of the transient absorption spectrum of ITI **8a** (red), **8b** (purple), **8c** (blue) and **8d** (black) in DCM on the TFA concentration as given in the upper *x*-axis, after nanosecond laser excitation. The lower *x*-axis displays the concentration of TFA monomers based on the association constant of 1.5 l mol^−1^ reported in ref. 34 (see Section S2 of the ESI† for further details). (b) Photoswitching of compound Z-**8d**-H^+^ (20 μM) in 3.26 M TFA solution in DCM (32.8 mM TFA monomer^34^) with green (*λ* = 526 nm) and red (*λ* = 652 nm) light (at 10 °C under stirring (900 rpm) conditions) (see Section S7 of the ESI† for further details).

To further experimentally investigate the validity of this theoretical model, we studied the influence of the acid titration on the kinetics of thermal relaxation for ITIs **8a–d** using nanosecond transient absorption spectroscopy. Fig. 7a displays the recovery half-lives of *E*-**8a–d** in DCM after nanosecond laser excitation as a function of the TFA concentration. It is gratifying to find that – in agreement with our model – this half-life is strongly dependent on the TFA concentration, showing an increase by more than two orders of magnitude for ITIs **8a**, **c** and **d**, and somewhat less for **8b**.

Although the changes in half-life are substantial, they differ from what one might *a priori* have expected on the basis of the calculated barriers for the direct back-isomerization of *E*-**8**-H^+^ to *Z*-**8**-H^+^ (step 3, Fig. 6), since these barriers would for all practical purposes lead to stable compounds *E*-**8**-H^+^. Our experiments indicate that the main reason that the observed half-lives are restricted to the order of tens of seconds is the self-association of TFA in DCM. As a result, dimers and higher-order clusters are formed at higher concentrations, and these clusters are not directly involved in the acid–base equilibrium (step 4, Fig. 6). In fact, the lower *x*-axis in Fig. 7a clearly indicates that the range of TFA monomer molarities that can effectively be employed is rather limited. Such a conclusion is supported by experiments in which the decay of the photostationary state reached after CW illumination was monitored for ITI solutions with high TFA concentrations employing steady-state UV-vis absorption spectroscopy (see below and Section S7†). In these experiments, the half-life was found to depend on whether the solution was stirred or not, with longer half-lives observed under stirring conditions. Moreover, a detailed global analysis of the recovery time shows a multi-exponential behavior, indicative of the presence of different species for which it is reasonable to assume that they are associated with dimers and higher-order clusters (see Section S7†). These observations are in line with a scenario in which restoring equilibrium (step 4, Fig. 6) after photoexcitation at high TFA concentrations is slowed down by the fact that photochemically generated *E*-**8**-H^+^ molecules are in a local environment with a relative concentration of TFA monomers orders of magnitude lower than that of TFA clusters. Restoring this equilibrium will at the same time influence the relative concentrations of the TFA monomer, dimer, and higher-order clusters, and thereby lead to a multi-exponential behavior.

In our model, it is implicitly assumed – when explaining the increase of the *E*-ITI half-life by protonation – that the rate at which the acid–base equilibrium is established is much faster than step 5 (Fig. 6) through which the non-protonated *E*-**8** is removed from this equilibrium. To confirm this assumption, we performed experiments for **8d** at TFA concentrations for which both the protonated and non-protonated forms were present roughly in equal amounts, and irradiation took place at wavelengths corresponding to either the maximum of *Z*-**8d**-H^+^ or of the *Z*-**8d** absorption band. In both cases the same half-life of the metastable *E*-form was found, implying that the distribution of *E*-**8d** and *E*-**8d**-H^+^ quickly reached an equilibrium, thereby confirming the assumption on the high reaction rate of step 4.

Finally, we have explored the overall switching ability of ITI **8d** by steady-state spectroscopy (Fig. 7b). We were delighted to see that at high TFA concentrations (corresponding to ∼33 mM concentration of the TFA monomer) the protonated ITI *Z*-**8d**-H^+^ readily responds to irradiation with green light (*λ* = 526 nm) reaching a high photostationary state as is apparent from the considerable drop in absorbance at *λ* = 532 nm. At the same time, the increase in absorbance at *λ* = 652 nm indicates the formation of metastable *E*-**8d**-H^+^. Under the employed experimental conditions (10 °C and stirring) the thermal half-life was found to be on the order of 1 min. Interestingly, we observed a significantly faster reverse switching using irradiation with red light (*λ* = 652 nm). We thus conclude that protonated ITI **8d**-H^+^ can be operated photochemically in both *Z* → *E* and *E* → *Z* directions using green/red light.

## Conclusions and outlook

The application of multi-stimuli responsive molecules offers access to advanced and sophisticated functional materials.^46–50^ However, their rational design is challenging due to the interplay of multiple stimuli-responses and requires detailed understanding of the underlying mechanistic principles at the molecular level. In this context, and inspired by various natural systems, we have explored here the acidochromism of a newly developed, visible-light responsive ITI photoswitch, focusing on the influence of protonation on its photochemical and functional properties.

Spectroscopic investigations revealed that protonation of *Z*-ITI leads to the emergence of a strong absorption band which can extend to over 600 nm, and the calculations attributed this band to the S_0_–S_1_ transition that becomes bright in protonated *Z*-ITIs. Irradiation at this band results in very fast (∼10 ps) switching to the metastable *E*-ITI isomer. Detailed theoretical studies of key stationary points on the excited-state and ground potential energy surfaces revealed that the ground-state acid–base equilibrium between *E*-**8**-H^+^ and *E*-**8** plays a key role in the operation of this switch. As a result of a very high barrier for thermal re-isomerization to the *Z*-**8**-H^+^ isomer, modulation of this acid–base equilibrium has been shown to offer an excellent means to actively tune the recovery time of the system for specific purposes. In fact, ITI switches are in this respect on par with other photochromic systems, in which the lifetime of the photochemically produced species can be tuned over several orders of magnitude.^15,21,22,51,52^ Importantly, we demonstrated that the protonated ITI **8**-H^+^ can be photochemically operated in both directions using low-energy green and red light, similar to the most advanced visible-light-operated P-type photochromes,^21,53–55^ albeit the relatively short half-life still remaining a challenge.

Altogether, protonation of ITI leads to red-shifted absorption maxima and longer half-lives, both of great importance for its applicability. However, in the present experiments, the tunability of the half-life has been limited by the employed solvents and acids. We are therefore presently exploring ITI derivatives able to operate under conditions in which this is no longer a limiting factor, also in aqueous solutions of importance for biological applications. In this context, we also recognize the challenges that stem from using protonation equilibria to reversibly manipulate the system with proton concentration, as it results in formation of side products after repeated cycles of acid and base addition. However, combining ITI switching with photochemical, reversible proton generation using a photoacid, as shown before for hydrazone switches, could offer a clean solution to this problem.^56^

In a broader perspective, the results presented here demonstrate that ITI shows clear functional similarities to visible-light-responsive azobenzenes,^14,15,52,57^ while featuring an obvious structural similarity to hemithioindigo^21,58^ photoswitches. This striking observation underlines the intriguing nature of ITI as a truly hybrid photoswitch and will hopefully inspire its future applications in designing visible-light-responsive molecular systems.

## Author contributions

M. W. H. H. and W. S. conceived the project and designed the molecules. All calculations were done by M. M. and A. D. L.; M. W. H. H. performed the synthesis. Nanosecond TA spectroscopy was performed by M. W. H. H., J. F., M. H. and W. J. B., while M. T. and M. D. D. performed the femtosecond TA spectroscopic experiments. Steady state UV-vis experiments were performed by M. W. H. H. and W. S. The manuscript was written by M. M., M. W. H. H., A. D. L., M. D. D., W. J. B. and W. S. The research was supervised by W. S., M. M., W. J. B., M. D. D. and B. L. F. All authors discussed the results and progress in all stages.

## Conflicts of interest

There are no conflicts to declare.

## Supplementary Material

SC-012-D0SC07000A-s001

## References

[cit1] Feringa B. L. and Browne W. R., Molecular Switches, Wiley-VCH, Weinheim, 2nd edn, 2011

[cit2] Pianowski Z. L. (2019). Chem. –Eur. J..

[cit3] Groppi J., Baroncini M., Venturi M., Silvi S., Credi A. (2019). Chem. Commun..

[cit4] Kay E. R., Leigh D. A. (2015). Angew. Chem., Int. Ed..

[cit5] Szymański W., Beierle J. M., Kistemaker H. A. V., Velema W. A., Feringa B. L. (2013). Chem. Rev..

[cit6] Ankenbruck N., Courtney T., Naro Y., Deiters A. (2018). Angew. Chem., Int. Ed..

[cit7] Welleman I. M., Hoorens M. W. H., Feringa B. L., Boersma H. H., Szymański W. (2020). Chem. Sci..

[cit8] Goulet-Hanssens A., Eisenreich F., Hecht S. (2020). Adv. Mater..

[cit9] Russew M. M., Hecht S. (2010). Adv. Mater..

[cit10] Garavelli M., Celani P., Bernardi F., Robb M. A., Olivucci M. (1997). J. Am. Chem. Soc..

[cit11] Habuchi S., Dedecker P., Hotta J.-i., Flors C., Ando R., Mizuno H., Miyawaki A., Hofkens J. (2006). Photochem. Photobiol. Sci..

[cit12] Luecke H., Schobert B., Richter H. T., Cartailler J. P., Lanyi J. K. (1999). Science.

[cit13] Velazquez Escobar F., Piwowarski P., Salewski J., Michael N., Fernandez Lopez M., Rupp A., Muhammad Qureshi B., Scheerer P., Bartl F., Frankenberg-Dinkel N., Siebert F., Andrea Mroginski M., Hildebrandt P. (2015). Nat. Chem..

[cit14] Samanta S., Babalhavaeji A., Dong M., Woolley G. A. (2013). Angew. Chem., Int. Ed..

[cit15] Dong M., Babalhavaeji A., Collins C. V., Jarrah K., Sadovski O., Dai Q., Woolley G. A. (2017). J. Am. Chem. Soc..

[cit16] Kennedy A. D. W., Sandler I., Andréasson J., Ho J., Beves J. E. (2020). Chem. –Eur. J..

[cit17] Roldan D., Cobo S., Lafolet F., Vilà N., Bochot C., Bucher C., Saint-Aman E., Boggio-Pasqua M., Garavelli M., Royal G. (2015). Chem. –Eur. J..

[cit18] Wolf J., Huhn T., Steiner U. E. (2015). Phys. Chem. Chem. Phys..

[cit19] Pu S. Z., Sun Q., Fan C.-B., Wang R.-J., Liu G. (2016). J. Mater. Chem. C.

[cit20] Liao Y. (2017). Acc. Chem. Res..

[cit21] Kink F., Collado M. P., Wiedbrauk S., Mayer P., Dube H. (2017). Chem. –Eur. J..

[cit22] Shao B., Aprahamian I. (2019). ChemPhotoChem.

[cit23] Berton C., Busiello D. M., Zamuner S., Solari E., Scopelliti R., Fadaei-Tirani F., Severin K., Pezzato C. (2020). Chem. Sci..

[cit24] Halbritter T., Kaiser C., Wachtveitl J., Heckel A. (2017). J. Org. Chem..

[cit25] Peters M. V., Stoll R. S., Kühn A., Hecht S. (2008). Angew. Chem., Int. Ed..

[cit26] Weston C. E., Richardson R. D., Fuchter M. J. (2016). Chem. Commun..

[cit27] Hoorens M. W. H., Medved’ M., Laurent A. D., Di Donato M., Fanetti S., Slappendel L., Hilbers M., Feringa B. L., Buma W. J., Szymanski W. (2019). Nat. Commun..

[cit28] Carrera E., Jones P. S., Iglesias S., Guadagno J. V., Warburton E. A., Fryer T. D., Aigbirhio F. I., Baron J. C. (2011). J. Cereb. Blood Flow Metab..

[cit29] Lerch M. M., Hansen M. J., Velema W. A., Szymanski W., Feringa B. L. (2016). Nat. Commun..

[cit30] Lehn J. M. (1970). Dyn. Stereochem. Fortschritte der Chem. Forschung.

[cit31] Lehn J. M. (2006). Chem. –Eur. J..

[cit32] Greb L., Eichhöfer A., Lehn J. M. (2015). Angew. Chem., Int. Ed..

[cit33] Suslova E. E., Ovchenkova E. N., Lomova T. N. (2014). Tetrahedron Lett..

[cit34] Christian S. D., Stevens T. L. (1972). J. Phys. Chem..

[cit35] Frisch M. J., Trucks G. W., Schlegel H. B., Scuseria G. E., Robb M. A., Cheeseman J. R., Scalmani G., Barone V., Mennucci B., Petersson G. A., Nakatsuji H., Caricato M., Li X., Hratchian H. P., Izmaylov A. F., Bloino J., Zheng G., Sonnenberg J. L., Hada M., Ehara M., Toyota K., Fukuda R., Hasegawa J., Ishida M., Nakajima T., Honda Y., Kitao O., Nakai H., Vreven T., Montgomery Jr J. A., Peralta J. E., Ogliaro F., Bearpark M., Heyd J. J., Brothers E., Kudin K. N., Staroverov V. N., Kobayashi R., Normand J., Raghavachari K., Rendell A., Burant J. C., Iyengar S. S., Tomasi J., Cossi M., Rega N., Millam J. M., Klene M., Knox J. E., Cross J. B., Bakken V., Adamo C., Jaramillo J., Gomperts R., Stratmann R. E., Yazyev O., Austin A. J., Cammi R., Pomelli C., Ochterski J. W., Martin R. L., Morokuma K., Zakrzewski V. G., Voth G. A., Salvador P., Dannenberg J. J., Dapprich S., Daniels A. D., Farkas Ö., Foresman J. B., Ortiz J. V., Cioslowski J. and Fox D. J., Gaussian 09 (Revision A.02), Gaussian Inc., Wallingford CT, 2009

[cit36] Frisch M. J., Trucks G. W., Schlegel H. B., Scuseria G. E., Robb M. A., Cheeseman J. R., Scalmani G., Barone V., Petersson G. A., Nakatsuji H., Li X., Caricato M., Marenich A. V., Bloino J., Janesko B. G., Gomperts R., Mennucci B., Hratchian H. P., Ortiz J. V., Izmaylov A. F., Sonnenberg J. L., Williams-Young D., Ding F., Lipparini F., Egidi F., Goings J., Peng B., Petrone A., Henderson T., Ranasinghe D., Zakrzewski V. G., Gao J., Rega N., Zheng G., Liang W., Hada M., Ehara M., Toyota K., Fukuda R., Hasegawa J., Ishida M., Nakajima T., Honda Y., Kitao O., Nakai H., Vreven T., Throssell K., Montgomery Jr J. A., Peralta J. E., Ogliaro F., Bearpark M. J., Heyd J. J., Brothers E. N., Kudin K. N., Staroverov V. N., Keith T. A., Kobayashi R., Normand J., Raghavachari K., Rendell A. P., Burant J. C., Iyengar S. S., Tomasi J., Cossi M., Millam J. M., Klene M., Adamo C., Cammi R., Ochterski J. W., Martin R. L., Morokuma K., Farkas O., Foresman J. B. and Fox D. J., Gaussian 16 (Revision A.03), Gaussian Inc., Wallingford CT, 2016

[cit37] Neese F. (2018). Wiley Interdiscip. Rev.: Comput. Mol. Sci..

[cit38] Neese F. (2012). Wiley Interdiscip. Rev.: Comput. Mol. Sci..

[cit39] TURBOMOLE V7.3, 2018, a development of University of Karlsruhe and Forschungszentrum Karlsruhe GmbH, 1989–2007, TURBOMOLE GmbH, since 2007, http://www.turbomole.com

[cit40] Zhao Y., Truhlar D. G. (2008). Theor. Chem. Acc..

[cit41] Ditchfield R., Hehre W. J., Pople J. A. (1971). J. Chem. Phys..

[cit42] Marenich A. V., Cramer C. J., Truhlar D. G. (2009). J. Phys. Chem. B.

[cit43] Riplinger C., Pinski P., Becker U., Valeev E. F., Neese F. (2016). J. Chem. Phys..

[cit44] Purvis G. D., Bartlett R. J. (1982). J. Chem. Phys..

[cit45] Raghavachari K., Trucks G. W., Pople J. A., Head-Gordon M. (1989). Chem. Phys. Lett..

[cit46] Nie H., Self J. L., Kuenstler A. S., Hayward R. C., Read de Alaniz J. (2019). Adv. Opt. Mater..

[cit47] Abdollahi A., Roghani-Mamaqani H., Razavi B. (2019). Prog. Polym. Sci..

[cit48] Zhuang J., Gordon M.
R., Ventura J., Li L., Thayumanavan S. (2013). Chem. Soc. Rev..

[cit49] Fihey A., Perrier A., Browne W. R., Jacquemin D. (2015). Chem. Soc. Rev..

[cit50] Schattling P., Jochum F. D., Theato P. (2014). Polym. Chem..

[cit51] Ludwanowski S., Ari M., Parison K., Kalthoum S., Straub P., Pompe N., Weber S., Walter M., Walther A. (2020). Chem. –Eur. J..

[cit52] Dong M., Babalhavaeji A., Hansen M. J., Kálmán L., Woolley G. A. (2015). Chem. Commun..

[cit53] Petermayer C., Thumser S., Kink F., Mayer P., Dube H. (2017). J. Am. Chem. Soc..

[cit54] Yang Y., Hughes R. P., Aprahamian I. (2014). J. Am. Chem. Soc..

[cit55] Zweig J. E., Newhouse T. R. (2017). J. Am. Chem. Soc..

[cit56] Tatum L. A., Foy J. T., Aprahamian I. (2014). J. Am. Chem. Soc..

[cit57] Samanta S., Beharry A. A., Sadovski O., McCormick T. M., Babalhavaeji A., Tropepe V., Woolley G. A. (2013). J. Am. Chem. Soc..

[cit58] Wiedbrauk S., Dube H. (2015). Tetrahedron Lett..

