# Supplementary material for: Tailoring the optical and dynamic properties of iminothioindoxyl photoswitches through acidochromism
Source: Chem Sci. 2021 Feb 9;12(12):4588–98. doi: 10.1039/d0sc07000a (PMC8179557; doi:10.1039/d0sc07000a)
Supplement: SC-012-D0SC07000A-s001 [file SC-012-D0SC07000A-s001.pdf]

Electronic Supplementary Material (ESI) for Chemical Science.
This journal is © The Royal Society of Chemistry 2021

Supporting Information for:

Tailoring the optical and dynamic properties of iminothioindoxyl photoswitches through acidochromism

Miroslav Medved',*[3,4,a] Mark W. H. Hoorens,[1,2,a] Mariangela Di Donato,[6,7] Adèle D. Laurent,[5] Jiayun Fan,[8] Maria Taddei,[6] Michiel Hilbers,[8] Ben L. Feringa,[2] Wybren Jan Buma,[8,9] Wiktor Szymanski*[1,2]

Table of contents:

S1. General methods S1
S1.1 Synthetic and analytical procedures S1
S1.2 Computational details S2
S1.3 Ultrafast spectroscopy S3
S1.4 Nanosecond transient absorption spectroscopy S4
S2. Association of TFA in DCM S5
S3. UV-Vis study on acid titrations S7
S4. NMR study on acid titrations S8
S5. Ultrafast spectroscopy S9
S6. Nanosecond transient absorption spectra S13
S7. Influence of TFA association on recovery dynamics S15
S8. Computational studies S17
S8.1 Adequateness of employed methods S17
S8.2 Acidity of Z-H+ and E-H+ S21
S8.3 Electronic transitions S22
S8.4 MO analysis of electronic transitions for protonated forms (ITI-H) S29
S8.5 MO analysis for neutral forms (ITI) S33
S8.6 Excited state structure of Z-H$^+$ and ESIPT S37
S8.7 Back-isomerization of protonated forms (Z-H+ to E-H+) S39
S8.8 Back-isomerization of neutral forms (E to Z) S39
S9. Bibliography S40

S1. General methods

S1.1 Synthetic and analytical procedures

The synthesis of ITI compounds was performed according to previously published procedures.[1] Nuclear Magnetic Resonance (NMR) spectra were recorded using an Agilent Technologies 400-MR (400/54 Premium Shielded) spectrometer (400 MHz), at room temperature (22–24 °C), unless indicated otherwise. All solutions for absorption spectra were prepared in Uvasol® grade solvents and were measured in quartz cuvettes with a 1 cm path-length.

S1.2 Computational details

Following our pilot study of ITIs,[1] the ground state (GS) structures of the *Z*/*E*-isomers and the GS transition states (TS) of the back isomerization (*E*→*Z*; thermal relaxation process) for both neutral ITIs (**8a-d**) and their protonated counterparts (**8a-d**-H^+) were optimized at the M06-2X level[2] using the 6-31+G(d) atomic basis set,[3] since this exchange-correlation functional is known to perform well not only for the GS thermochemistry, but also in describing excited states (see also section S8.1).[4] All minima were checked against the presence of imaginary frequencies, whereas the TS were confirmed to be the first-order saddle points exhibiting a single imaginary frequency. The solvent effects were considered employing the Solvation Model based on Density (SMD).[5] The parameters for dichloromethane (DCM) were used consistently with the experimental procedure. Using the DFT geometries, the GS energies of **8a** were also calculated by the spin-component-scaled second-order Møller–Plesset perturbation theory,[6] using the resolution of identity approximation[7] (RI-SCS-MP2) and the domain-based local pair natural orbital (DLPNO)[8] coupled cluster with singles and doubles and perturbative triples (CCSD(T))[9] method combined with the SMD model, applying the TightPNO settings as defined in ref.[10] with the cc-pVTZ basis set.[11] The latter is hereafter referred to as the composite DLPNO-CCSD(T)/M06-2X/SMD approach.

To determine the activation barrier for the back-isomerization of *E*-**8**-H^+ to *Z*-**8**-H^+, a relaxed scan for GS torsional rotation of **8**-H^+ was performed using the M06-2X/6-31+G(d)/UHF/Guess(INDO,Mix,always) setup in the Gaussian calculations. In the next step, the structure with the maximum energy as obtained from the scan was used in TS optimization (using the same setup) to get the true GS TS structures. The activation barrier for the back-isomerization

of neutral ITIs in DCM was determined by TS optimizations, starting from TS structures obtained at the same level of theory in chloroform (published in ref.[1]). The suitability of the application of the standard DFT approach in both neutral and protonated cases was checked by the spin-flip TD-DFT (SF-TD-DFT) calculations using the GAMESS program, ver. R1,[12] employing the BHHLYP functional[13] in combination with the 6-31++G(d,p) basis set and the SMD solvation model. SF-TD-DFT is an extension of DFT to situations in which significant non-dynamical correlation is present, i.e., it is suitable for semi-quantitative analysis of systems in the vicinity of a conical intersection. The method is based on the spin–flip (SF) approach which is capable of describing multireference wave functions within a single reference formalism as spin–flipping, i.e., $\alpha \rightarrow \beta$, excitations from a high-spin (M_s=1) triplet reference state.[14]

Vertical excitation energies (VEE) were obtained with a larger basis set (6-311++G(2df,2p)). The SMD was combined with the corrected linear response (cLR) approach[15] to account, state-specifically, for the solvent polarization response upon absorption. The gas phase VEEs obtained with M06-2X were compared to the algebraic diagrammatic construction through second order (ADC(2)) and to the linear-response second-order coupled-cluster (CC2)[16] data for **8a**. Geometry optimization of the S_1 state structure of *Z*-**8a**-H^+, relaxed scans for the excited-state intramolecular proton transfer (ESIPT) process and the ES isomerization were performed at the TD-M06-2X/6-31+G(d)/SMD(DCM) level of theory. For the latter, the SF-TD-DFT calculations were also carried out to check the appropriateness of the LR-TD-DFT approach.

All (TD)-DFT calculations were performed using the Gaussian09[17] and Gaussian16[18] programs. The Gaussian default thresholds and algorithms were used except for improving optimization. In the latter case, a threshold of 10^{-5} a.u. on average residual forces was imposed, a self-consistent field convergence criterion of 10^{-10} a.u., and the ultrafine DFT integration grid was used. The RI-SCS-MP2 and DLPNO-CCSD(T) calculations were carried out using the Orca program (ver. 4.2.1).[19,20] The CC2 and ADC(2) data were obtained with Turbomole (ver. 7.3).[21]

S1.3 Ultrafast spectroscopy

Ultrafast transient absorption spectra of protonated and neutral compounds **8a**, **8c** and **8d** were measured on a system consisting of a home-made Ti:sapphire laser oscillator and a regenerative amplifier system (Amplitude Pulsar), which produced 80 femtoseconds pulses at 810 nm with an

average output power of 450 to 500 mW. Excitation pulses at 400 nm where obtained by second harmonic generation of the fundamental laser output in a 2 mm thick BBO crystal. Pump pulses at 460-515 nm were obtained by pumping a commercial optical parametric amplifier system (TOPAS, Light Conversion). For all measurements, the pump beam polarization was set at magic angle with respect to the probe beam by rotating a λ/2 plate, so as to exclude rotational contributions to the transient signal.

The excitation powers were on the order of 50 to 100 nJ. The probe pulses were generated by focusing a portion of the 800 nm radiation beam on a 3 mm thick Calcium Fluoride window. The time delay between pump and probe pulses was introduced by sending the portion of 800 nm light used for probe generation though a motorized stage. After passing through the sample, the white light probe was sent to a flat field monochromator coupled to a home-made CCD detector. All measurements were performed in a quartz cell (2 mm thick) mounted on a movable stage in order to refresh the solution and avoid undesired photochemical degradation of the sample.

Analysis of the transient data was performed using Singular Value Decomposition (SVD)[22] and global analysis,[23] using the Glotaran software.[24] Global analysis allows the simultaneous fit at all the measured wavelengths with a combination of exponential decay functions and retrieves the kinetic constants describing the dynamic evolution of the system and the corresponding spectral components, called Evolution Associated Difference Spectra (EADS). A linear decay kinetic scheme was employed for data analysis.

S1.4 Nanosecond transient absorption spectroscopy

Nanosecond transient absorption spectra were recorded with an in-house assembled setup. Excitation was performed using a tunable Nd:YAG-laser system (NT342B, Ekspla) comprising a pump laser (NL300) with harmonics generators (SHG, THG) producing 355 nm to pump an optical parametric oscillator (OPO) with SHG connected in a single device. This laser system was operated at a repetition rate appropriate for the recovery time of the investigated sample, that is, at least lower than the inverse of five times the observed recovery time, and with a maximum repetition rate of 5 Hz. The probe light was generated by a high-stability short arc xenon flash lamp (FX-1160, Excelitas Technologies) using a modified PS302 controller (EG&G). This flash lamp was either operated at double the repetition rate of the excitation laser, or in bursts of shots separated by 40 ms and with a delay ranging from 0 to 39 ms with respect to the excitation laser

pulse for laser repetition rates lower than 2 Hz. Using a 50/50 beam splitter, the probe light was split equally into a signal beam and a reference beam and focused on the entrance slit of a spectrograph (SpectraPro-150, Princeton Instruments). The probe beam (A = 1 mm^2) was passed through the sample cell and orthogonally overlapped with the excitation beam on a 1 mm × 1 cm area. The excitation energy was recorded by measuring the excitation power at the back of an empty sample holder. In order to correct for fluctuations in the flash lamp spectral intensity, the reference was used to normalize the signal. Both beams were recorded simultaneously using a gated intensified CCD camera (PI-MAX3, Princeton Instruments) which has an adjustable gate of minimal 2.9 ns. A delay generator (DG535, Stanford Research Systems, Inc.) was used to time the excitation pulse, the flash lamp, and the gate of the camera. The setup was controlled by an in-house written LabView program.

Transient absorption spectra were globally analyzed, that is, fitting the time evolution of the data at all wavelengths simultaneously, using the Glotaran software.[24] Such analyses showed that the data could well be fitted with a single exponential decay function.

S2. Association of TFA in DCM

It is well known that carboxylic acids dissolved at high concentrations in nonpolar solvents may form dimers and higher order clusters. As a result, the concentration of TFA monomers available to protonate ITI switches can be significantly lower than the TFA concentration as calculated from the volumes of TFA and DCM used to make the solution. This, in turn, may have serious consequences for the interpretation of the dependence of the spectroscopy and dynamics of these switches on the TFA concentration. Although the association constant of TFA in DCM has not been reported yet, a value of (1.5±0.4 l·mol-1) has been reported for TFA in 1,2-dichloroethane.[25] Using such a constant, one rapidly comes to the conclusion that for TFA molarities higher than roughly 0.1 mM the molarity of TFA monomers is significantly lower than intended. Indeed, IR spectra of 0.64 M solutions of TFA in DCM, as used in the femtosecond transient absorption experiments (Figure S2.1), clearly show two bands in the carbonyl stretch region that derive from the TFA monomer and dimer, and a less intense third band at a lower frequency that might be associated with higher-order clusters. In view of the high TFA concentrations used in the present study we have therefore chosen to report both the total TFA molarities and TFA monomer molarities calculated based on an association constant of 1.5 l·mol-1.

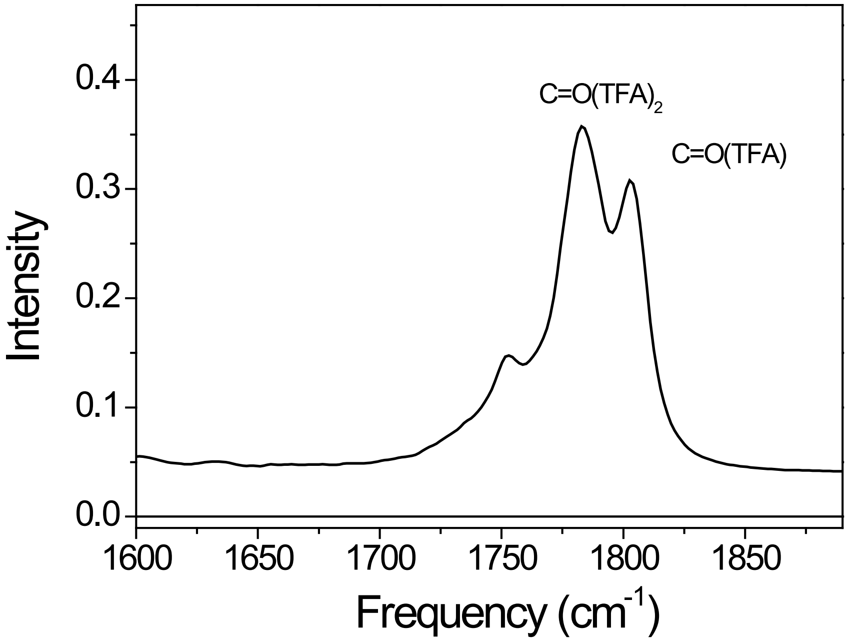

Figure S2.1 IR spectra of 0.64 M TFA in DCM (14.4 mM TFA monomers), carbonyl absorption region

S3. UV-Vis study on acid titrations

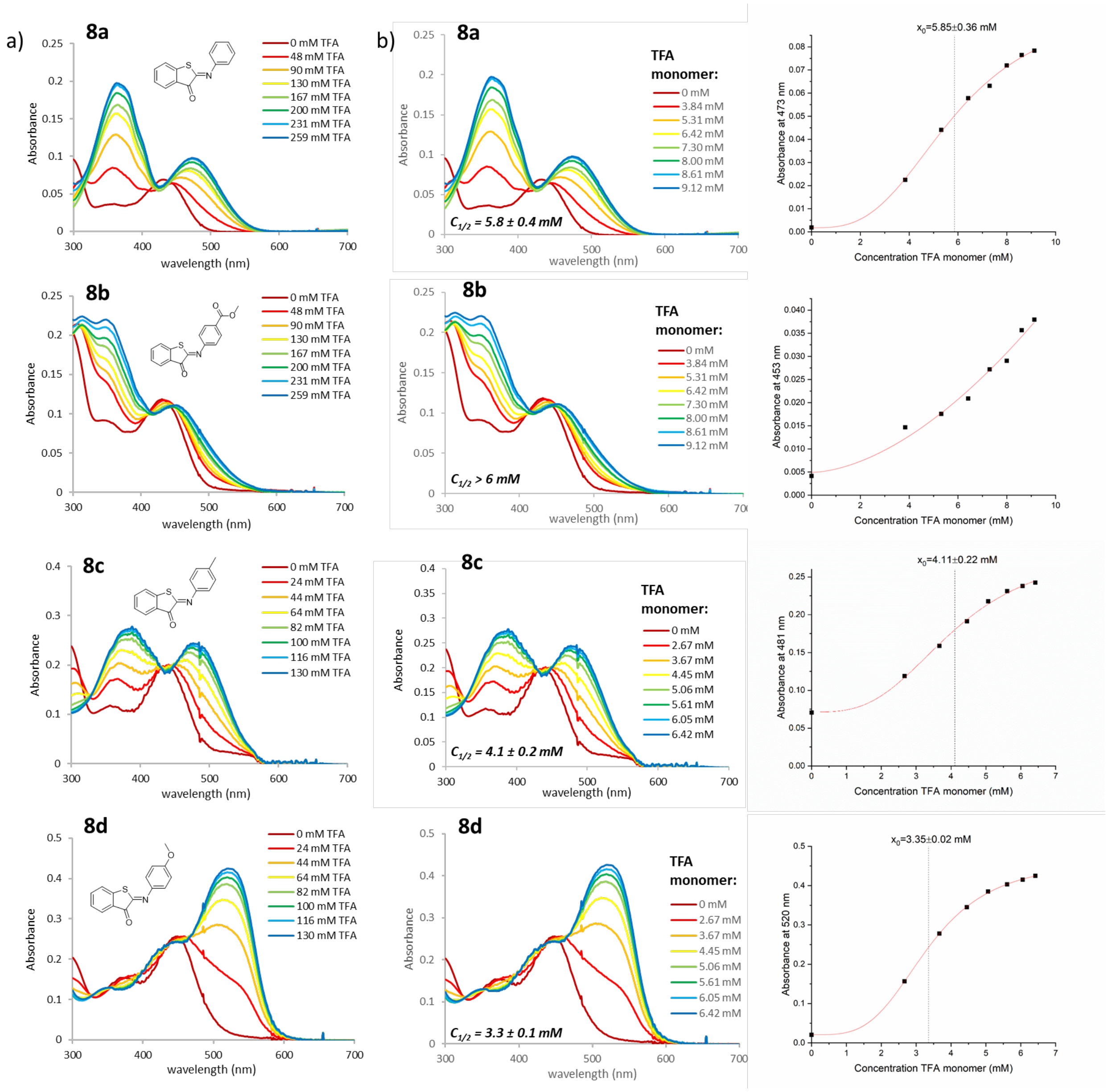

Figure S3.1 Titration curves for titration of compounds **8a-d** (20 μM) with total TFA concentrations (a), and monomer TFA concentrations (b) in DCM. Fitting of the absorbance changes is presented in (c). Red lines represent fits to a logistic function with x_0 being the infliction point. **8b** cannot be fitted with such a function; in this case the red line serves as a guide to the eye. The SD values represent the uncertainty of experimental point fitting.

S4. NMR study on acid titrations

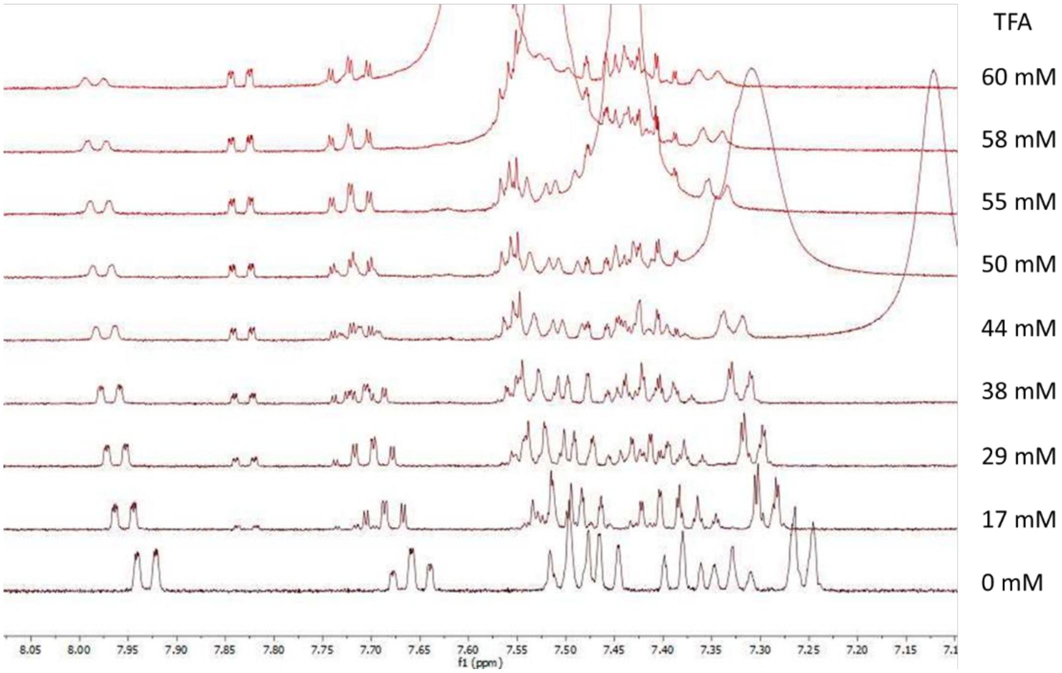

Figure S4.1 ^{1}H NMR titration of ITI **8a** in CD_2Cl_2 (0.5 mg/mL) with TFA (total concentration given).

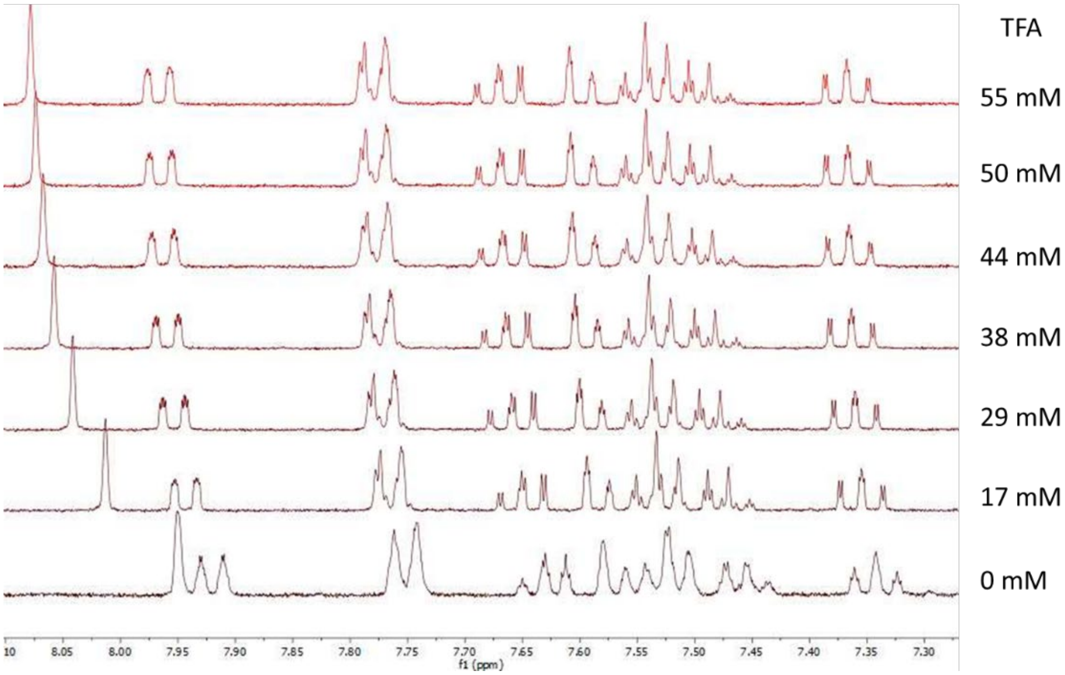

Figure S4.2 ^{1}H NMR titration of hemithioindigo **9** in CD_2Cl_2 (0.5 mg/mL) with TFA (total concentration given).

S5. Ultrafast spectroscopy

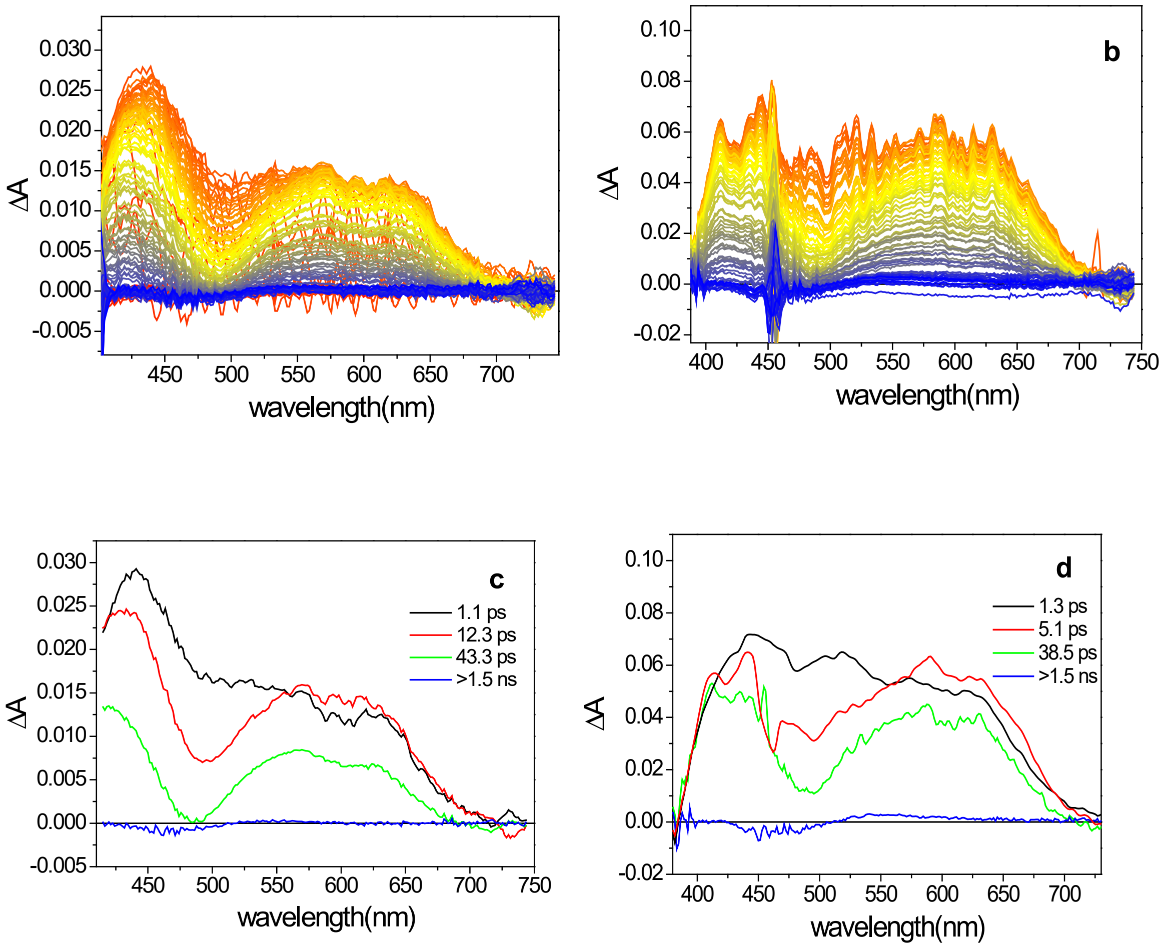

Figure S5.1 Transient absorption spectra (panels a, b) and EADS obtained from global analysis of data recorded for **8a-H**$^{+}$ in 0.64 M TFA in DCM (14.4 mM TFA monomers) upon excitation at 400 nm (left column) and 460 nm (right column).

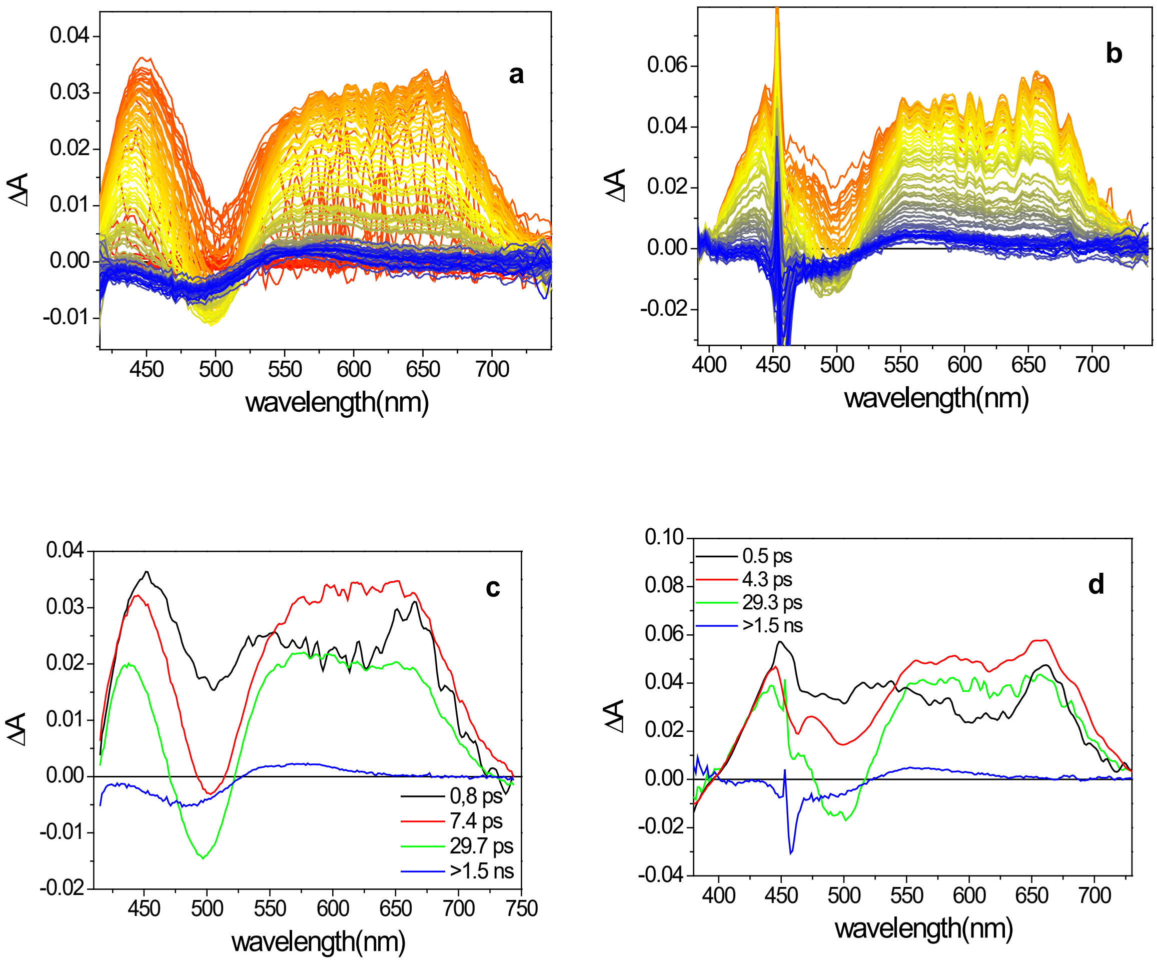

Figure S5.2 Transient Absorption spectra (panels a, b) and EADS obtained from global analysis of transient data recorded for **8c-H**$^+$ in 0.64 M TFA in DCM (14.4 mM TFA monomers) upon excitation at 400 nm (left column) and 460 nm (right column).

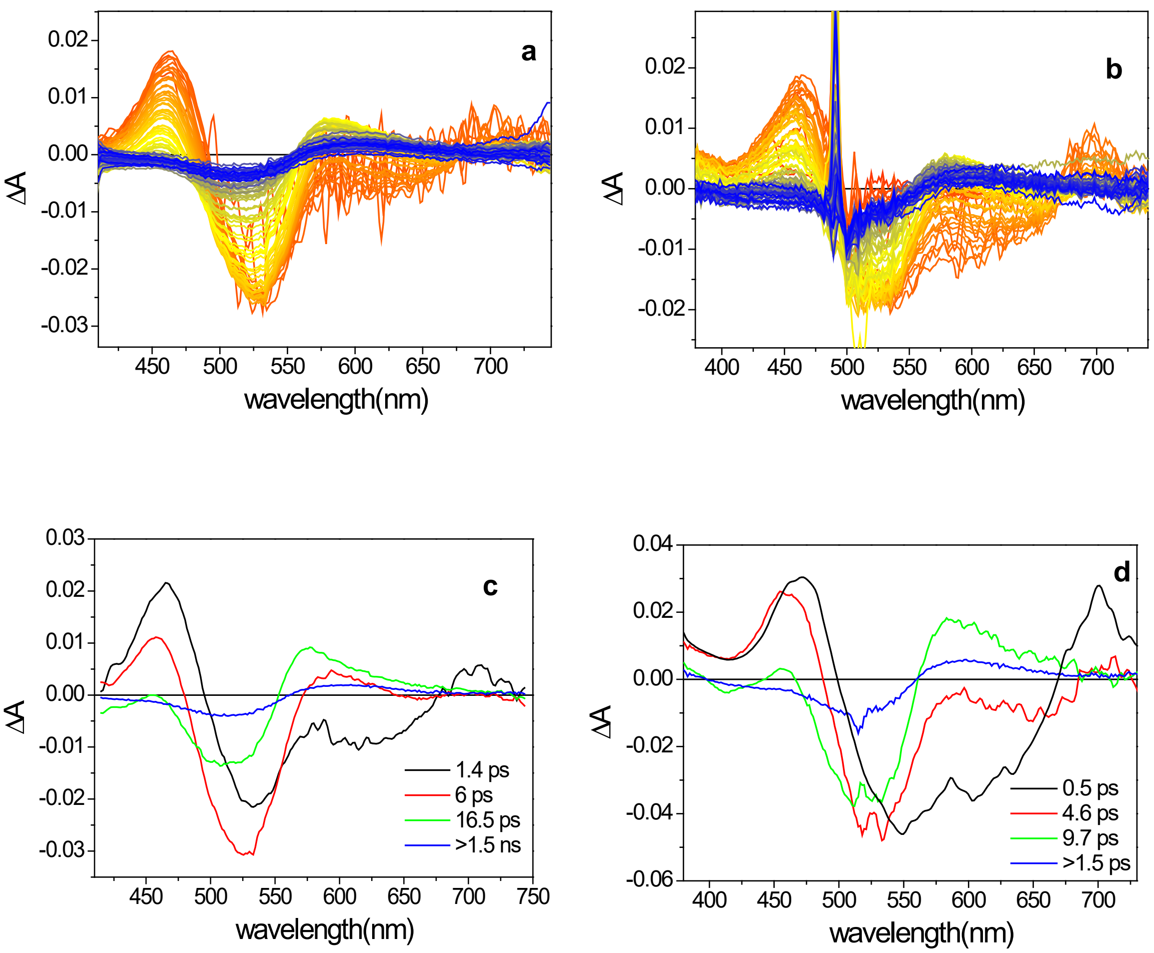

Figure S5.3 Transient Absorption spectra (panels a, b) and EADS obtained from global analysis of transient data recorded for **8d-H**$^{+}$ in 0.64 M TFA in DCM (14.4 mM TFA monomers) upon excitation at 400 nm (left column) and 514 nm (right column).

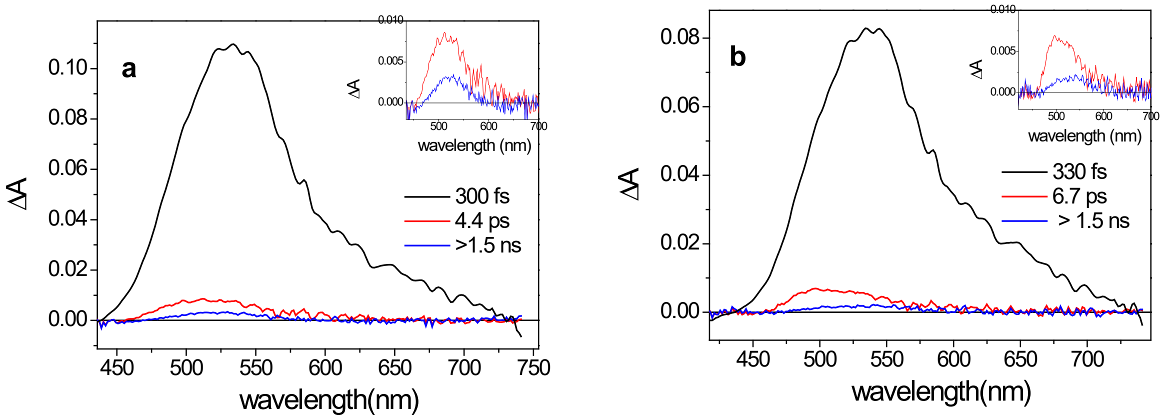

Figure S5.4 EADS obtained from global analysis of transient data recorded for compounds **8a** (panel a) and **8c** (panel b) in DCM upon excitation at 400 nm. The second and third EADS are magnified in the inset graph.

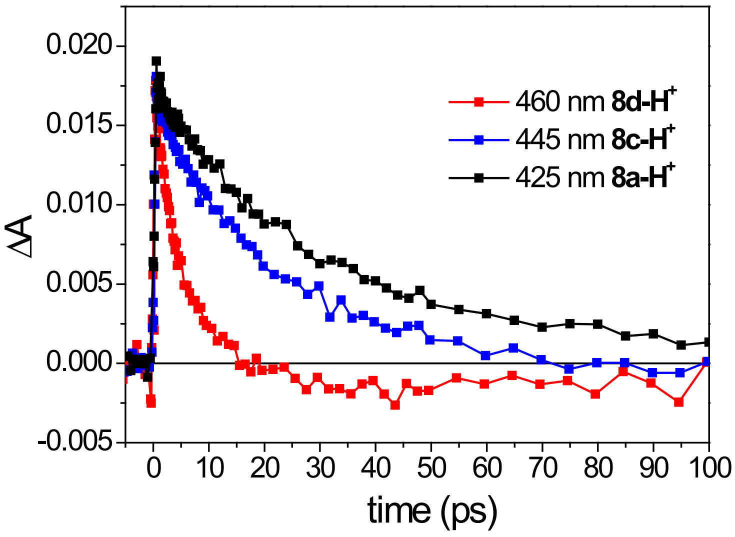

Figure S5.5 Comparison of selected kinetic traces registered for **8a-H**$^+$, **8c-H**$^+$ and **8d-H**$^+$ (0.64 M TFA in DCM, 14.4 mM TFA monomers) upon 400 nm excitation.

S6. Nanosecond transient absorption spectra

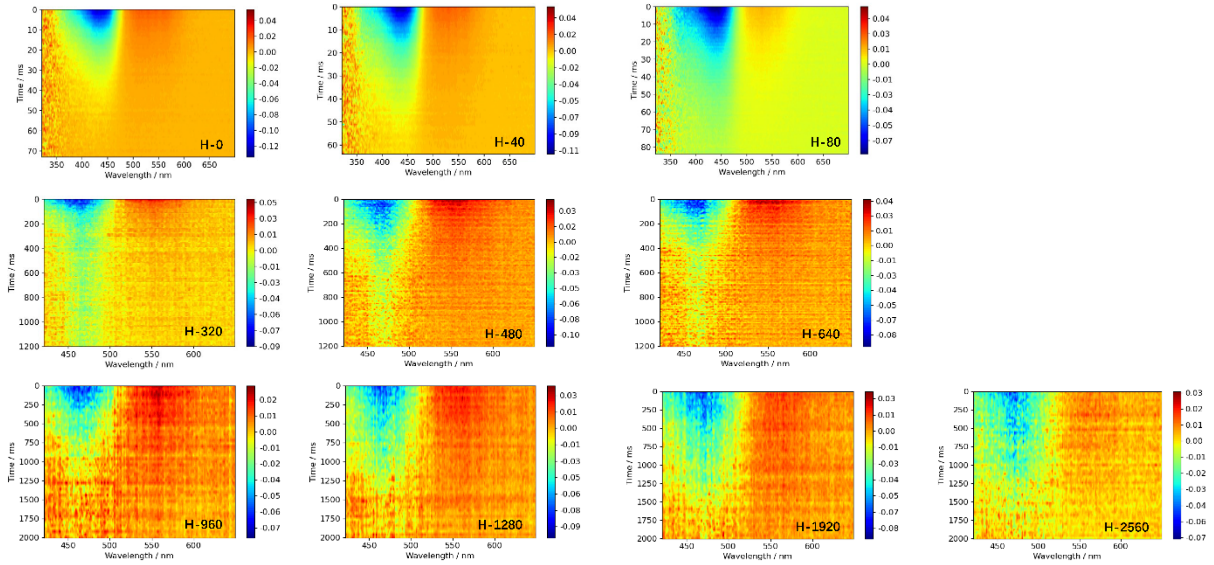

Figure S6.1 Transient absorption spectra obtained for **8a** and **8a-H$^+$** in solutions of TFA in DCM. Labels **x** in the **H-x** label in the lower right corner of each spectrum indicate the total molarity of TFA in mM. Notice that different rows of spectra have a different time axis.

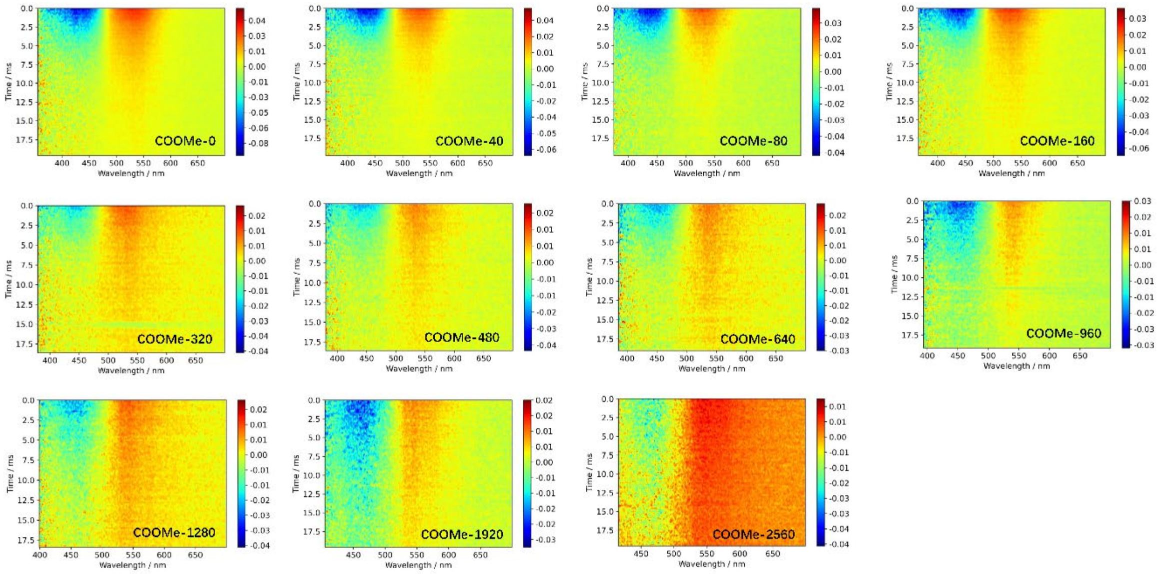

Figure S6.2 Transient absorption spectra obtained for **8b** and **8b-H$^+$** in solutions of TFA in DCM. Labels **x** in the **H-x** label in the lower right corner of each spectrum indicate the total molarity of TFA in mM. In this case all spectra have been plotted with the same time axis.

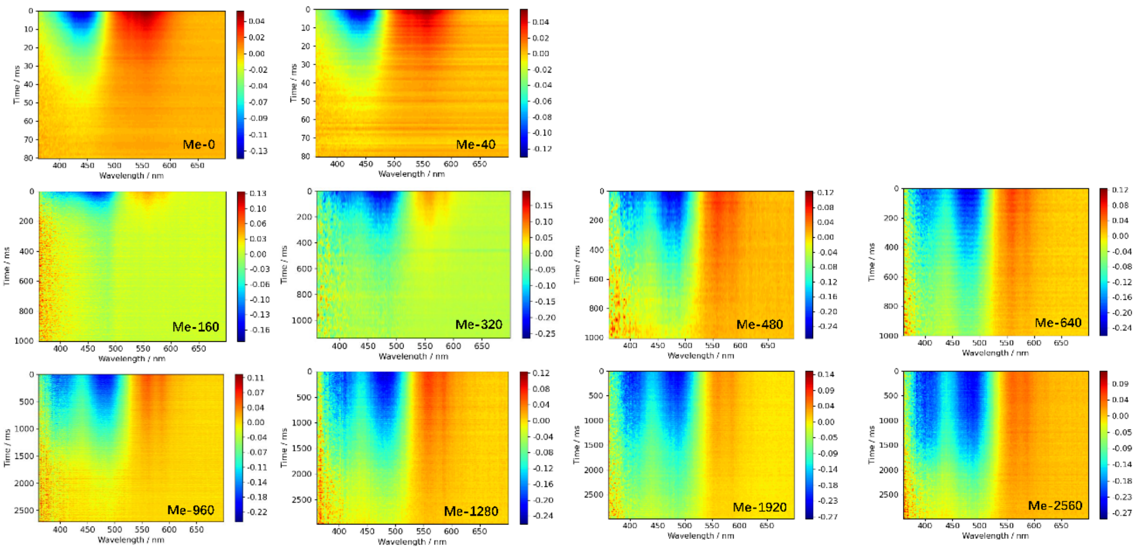

Figure S6.3 Transient absorption spectra obtained for **8c** and **8c-H**$^+$ in solutions of TFA in DCM. Labels **x** in the **H-x** label in the lower right corner of each spectrum indicate the total molarity of TFA in mM. Notice that different rows of spectra have a different time axis.

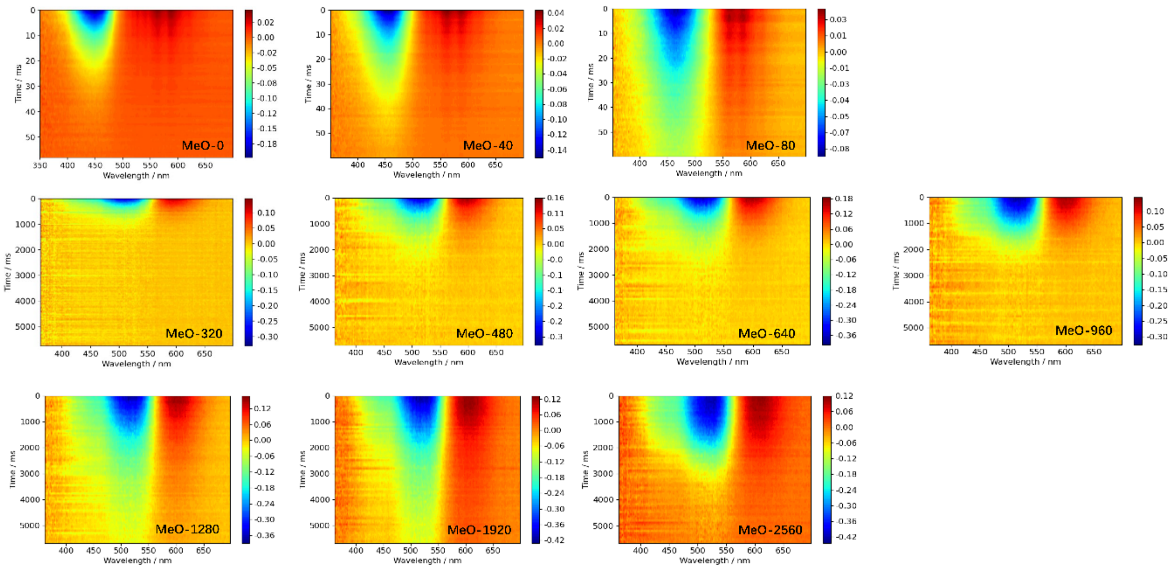

Figure S6.4 Transient absorption spectra obtained for **8dc** and **8d-H**$^+$ in solutions of TFA in DCM. Labels **x** in the **H-x** label in the lower right corner of each spectrum indicate the total molarity of TFA in mM. Notice that the first row of spectra has a different time axis than the second and third rows.

S7. Influence of TFA association on recovery dynamics

In our studies, photoswitching dynamics have been studied using two different approaches. In the first approach, nanosecond laser excitation was used to photoisomerize ITI-H^+ switches while the subsequent recovery dynamics were monitored using transient absorption. For high TFA concentrations, the half-lives turned out to be long enough that also a second approach based on irradiation with CW light at a specific wavelength could be employed. In this case, irradiation was used to reach a photostationary state after which irradiation was stopped. Both the build-up and the decay of the photostationary state were followed by monitoring the absorption spectrum. Initially, we found that the half-lives determined using the CW approach were substantially longer than the ones determined with nanosecond transient absorption.

The fundamental difference between the two experiments is that in the CW experiments the solution could be stirred, while the transient absorption experiment does not allow to do so since stirring removes the photoexcited molecules from the volume that is probed with the probe light. To determine whether stirring is indeed the cause for the longer half-lives measured with the CW approach, we performed CW measurements on *Z*-**8d**-H^+ (20 μM) in a 3.26 M total TFA solution with and without stirring. Inspection of the kinetic traces obtained at Figure S7.1a at the maxima of the *Z* and *E* isomers shows that stirring indeed leads to longer half-lives, which is also confirmed by global analyses of the two experiments (Figure S7.1b and c). We therefore conclude that restoring equilibrium I-4 (see Figure 7 of the manuscript) after photoexcitation at high TFA concentrations is slowed down by the fact that photochemically generated *E*-**8d**-H^+ molecules are in a local environment with a relative concentration of TFA monomers orders of magnitude lower than that of TFA clusters.

Interestingly, we find that the global analyses also lead to the conclusion that the decay dynamics exhibit a multi-exponential behaviour. We tentatively attribute this behaviour to the presence of not only TFA dimers but also higher-order clusters. Restoring equilibrium I-4 will in that case also influence the dimer and higher-order association equilibria. Comparing the faster decay time under stirring conditions with the decay time found in the nanosecond transient absorption experiments we find a reasonable agreement between the two. The slower decay time, on the other hand, is not observed in the latter experiments, but this is quite reasonable since on those timescales also diffusion processes will start to influence the measurement. The final very

long lifetime found with global analysis has a fixed value and has been added only to improve the convergence of the fit.

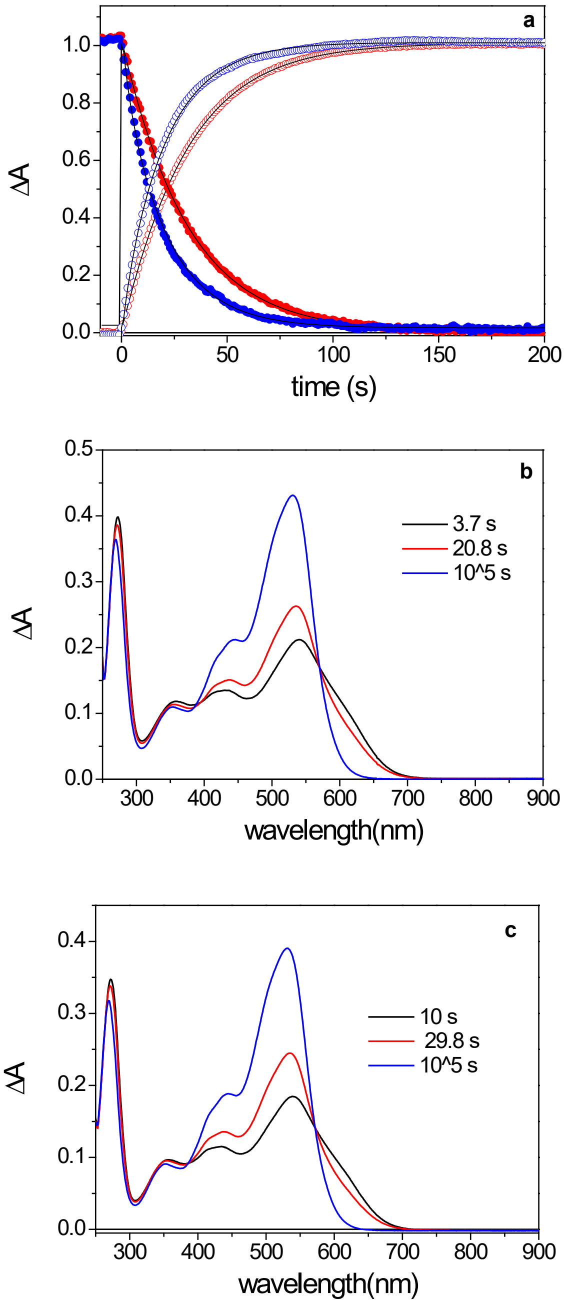

Figure S7.1 Influence of stirring on recovery dynamics of **8d**-H^+ isomers after reaching the photostationary state using CW irradiation at 526 nm. (a) Kinetic traces obtained at 526 (open symbols) and 652 nm (closed symbols) without stirring (red) and with stirring at 900 rpm (blue). Traces have been normalized. Panels (b) and (c) display global analyses of the time evolution of the absorption spectra without and with stirring.

S8. Computational studies

S8.1 Adequateness of employed methods

The accuracy of a specific DFT method is dependent on the target property and can be estimated by a thorough validation on large database sets against experimental and highly accurate theoretical (usually CCSD(T)/CBS) values. Such an assessment was presented by Mardirossian and Head-Gordon[26] showing that the M06-2X functional applied in our study belongs (together with MN15 and ωB97M-V) to the best performing ones exhibiting an uncertainty (in terms of RMSDs) of isomerization energies and energy barriers of 0.5 and 2.57 kcal/mol, respectively. To support the reliability of M06-2X in the present case, we performed RI-SCS-MP2 and DLPNO-CCSD(T)/cc-pVTZ/SMD calculations of the electronic relative energies and energy barriers for compound **8a** using the M06-2X geometries. These calculations show that the differences are indeed of expected magnitudes, with a maximum deviation (-3.3 kcal/mol) for the thermal back-isomerization of neutral **8a** (see Table S8.1). Concerning the SMD continuum model, its good performance for thermochemistry (including charged species) was reported by D.G. Truhlar and collaborators.[5] It should also be emphasized that DCM (the solvent considered in our study) belongs to polar aprotic solvents for which the application of implicit solvation models is generally considered to be appropriate.[27]

Concerning the application of M06-2X for the excited state calculations, the assessment of various DFT methods on large datasets involving different types of systems and transitions by Laurent and Jacquemin[4] revealed that -for single-reference systems and excited states with negligible double-excitation character (which is indeed the case for ITI(-H) as revealed by the %τ_2 (CC2) value[28] being ≤15%)- M06-2X belongs (together with RSH ωB97X-D and CAM-B3LYP functionals) to the best performing functionals, thus enabling -taking into account its performance in GS calculations- a treatment of both GS and ES of ITIs on equal footing. A good performance of M06-2X specifically in the case of non-protonated ITIs was presented in our previous work,[1] in which the VEE and oscillator strengths for the three lower electronically excited states (in the gas phase) were compared with CC2 and ADC(2) data. For protonated ITIs, we made a similar comparison confirming that M06-2X also performs very well in this case (Table S8.4). It is worth noting that both M06-2X VEE values for the $S_0 \rightarrow S_1$ and $S_0 \rightarrow S_3$ transitions are blue-shifted by 0.24 and 0.32 eV with respect to those obtained by CC2 and

ADC(2), respectively, which to a large extent rationalizes the necessity to red-shift (by 0.35 eV) the simulated spectra in Fig. 5.

GS structures of the Z/E forms and TSa of protonated and neutral 8a-d

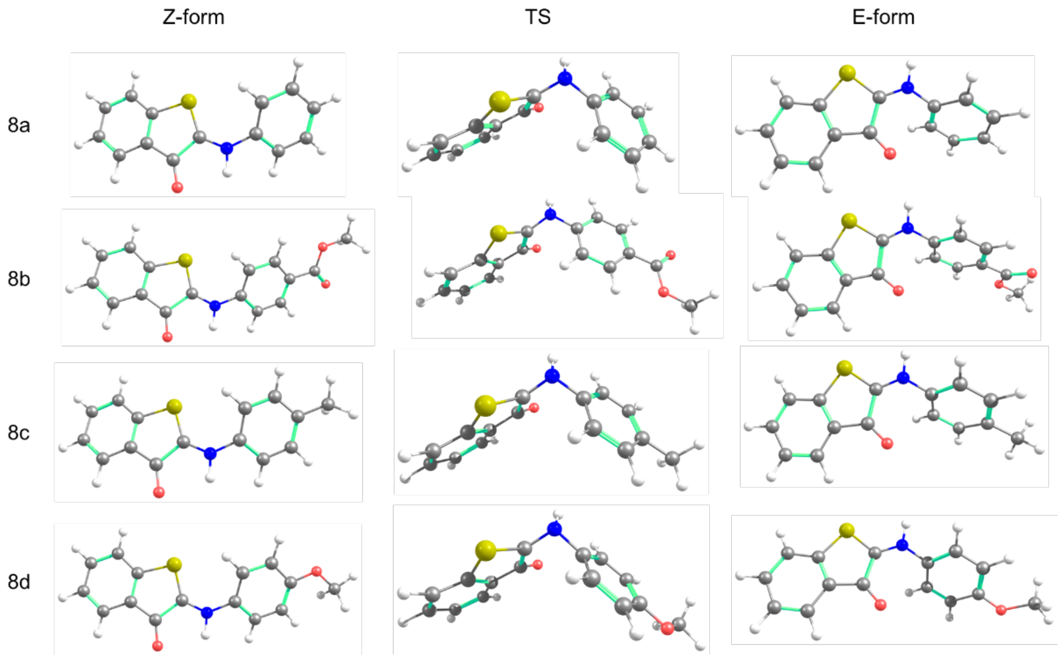

Figure S8.1 GS structures of **8-H**$^+$ in DCM optimized at the M06-2X/6-31+G(d)/SMD(DCM) level.

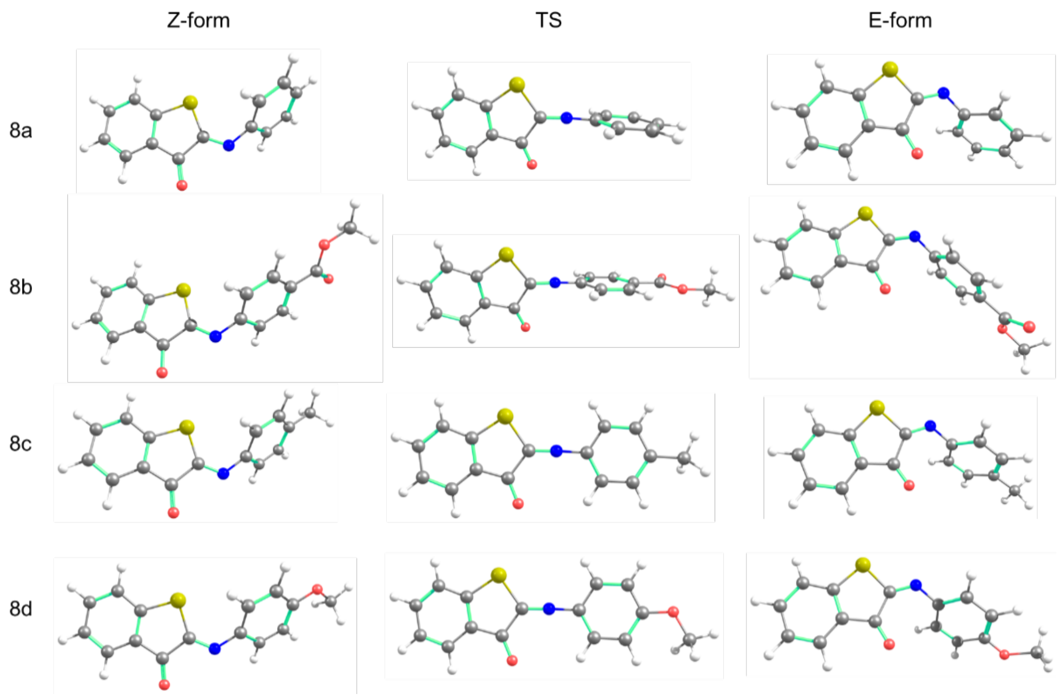

Figure S8.2 GS structures of **8** in DCM optimized at the M06-2X/6-31+G(d)/SMD(DCM) level.

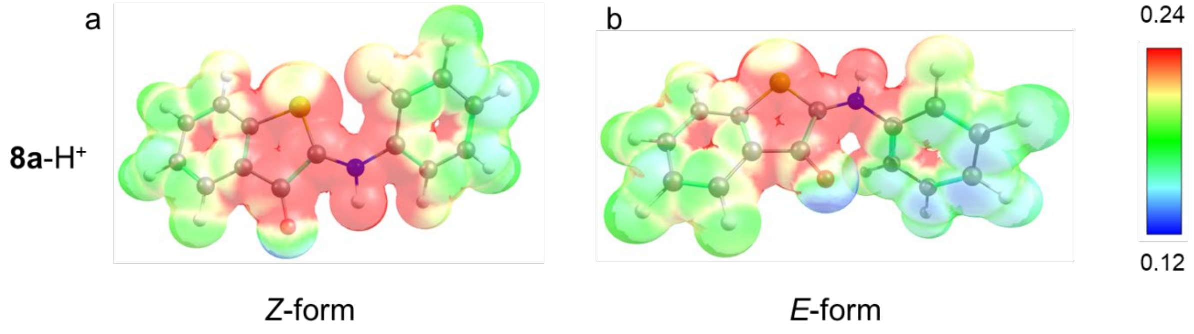

Figure S8.3 Electrostatic potential mapped spheres within the given range (in au) indicating the most positively charged regions (in red) in the *Z*-form (a) and *E*-form (b) of **8a**-H$^+$ (M06-2X/6-31+G(d)/SMD level of theory).

Table S8.1 The GS relative energies and Gibbs energies (kcal/mol, T = 298.15 K) of the E, Z and TS forms of ITI and ITI-H$^+$ (**8a-d**) in DCM calculated at the M06-2X/6-31+G(d)/SMD(DCM) level of theory. The barriers correspond to back isomerization (E to Z). The RI-SCS-MP2 / DPLNO-CCSD(T) electronic energy values obtained using the SMD solvation model and the cc-pVTZ basis set for **8a** are reported in parentheses. The corresponding Gibbs energies were obtained by adding the thermal and entropic contributions from the DFT calculation.

	R	ITI		ITI-H$^+$		ITI		ITI-H$^+$	
		$\Delta E_{Z\to E}$	$\Delta E^{\#}_{E\to Z}$	$\Delta E_{Z\to E}$	$\Delta E^{\#}_{E\to Z}$	$\Delta G_{Z\to E}$	$\Delta G^{\#}_{E\to Z}$	$\Delta G_{Z\to E}$	$\Delta G^{\#}_{E\to Z}$
**8a**	H	4.4<br>(3.9/3.9)	13.4<br>(16.0/16.7)	6.8<br>(6.5/6.2)	32.2<br>(31.7/31.1)	3.4<br>(2.9/2.9)	13.3<br>(15.9/16.6)	6.2<br>(5.9/5.6)	31.1<br>(30.6/30.0)
**8b**	COOMe	3.5	12.2	6.3	30.9	3.8	12.1	5.0	30.6
**8c**	Me	4.6	13.2[a]	7.1	32.6	3.6	13.7[a]	5.4	32.8
**8d**	MeO	4.7	12.3[a]	7.2	33.3	4.5	10.8[a]	7.6	32.1

[a] *The TS structure is planar.*

Table S8.2 Dihedral angles $\theta_1(C_1C_2N_4C_5)$ and $\theta_2(C_2N_4C_5C_6)$ (see Figure 5b for the numbering of atoms), cumulated Mulliken charges on the phenyl ring and the dipole moments (in Debye) of the Z and E forms of ITI and ITI-H$^+$ (**8a-d**) in DCM calculated at the M06-2X/6-31+G(d)/SMD(DCM) level of theory.

		ITI						ITI-H$^+$			
		Z form			*E* form			*Z* form		*E* form	
	R	θ_1/θ_2	q(Ph)	μ_{GS}	θ_1/θ_2	q(Ph)	μ_{GS}	θ_1/θ_2	q(Ph)	θ_1/θ_2	q(Ph)
**8a**	H	179.8/<br>51.7	0.10	5.63	9.4/<br>64.0	-0.04	4.80	178.4/<br>28.0	0.59	13.1/<br>55.9	0.35
**8b**	COOMe	179.6/<br>59.7	-0.05	7.41	1.08/<br>91.0	-0.16	6.93	179.2/<br>28.4	0.62	11.9/<br>58.6	0.36
**8c**	Me	180.0/<br>49.3	0.20	5.58	10.2/<br>60.8	0.05	4.18	177.6/<br>22.6	0.70	13.9/<br>53.8	0.49
**8d**	MeO	179.2/<br>36.6	0.36	4.33	12.4/<br>49.6	0.20	2.66	178.4/<br>21.4	0.71	16.6/<br>45.2	0.66

S8.2 Acidity of Z-H$^+$ and E-H$^+$

To estimate the pK_a values of Z and E forms of **8**-H$^+$ in water, the following equilibria were considered:

$$Z\text{-}\mathbf{8}\text{-H}^+ \text{ (sol)} \rightleftarrows Z\text{-}\mathbf{8} \text{ (sol)} + \text{H}^+ \text{ (sol)} \quad (1)$$

$$E\text{-}\mathbf{8}\text{-H}^+ \text{ (sol)} \rightleftarrows E\text{-}\mathbf{8} \text{ (sol)} + \text{H}^+ \text{ (sol)} \quad (2)$$

$$pK_a(\text{X-H}^+) = \frac{\triangle G_{sol}}{2.303RT} = \frac{G^*_{sol}(\text{H}^+) + G_{sol}(X) - G_{sol}(X\text{-H}^+)}{2.303RT} \quad (3)$$

$$\Delta\, pK_a(E - Z) = pK_a(E\text{-}\mathbf{8}\text{-H}^+) - pK_a(Z\text{-}\mathbf{8}\text{-H}^+) =$$

$$= \frac{G^*_{sol}(\text{H}^+) + G_{sol}(E) - G_{sol}(E\text{-}\mathbf{8}\text{-H}^+)}{2.303RT} - \frac{G^*_{sol}(\text{H}^+) + G_{sol}(Z) - G_{sol}(Z\text{-}\mathbf{8}\text{-H}^+)}{2.303RT} =$$

$$= \frac{G_{sol}(E) - G_{sol}(E\text{-}\mathbf{8}\text{-H}^+)}{2.303RT} - \frac{G_{sol}(Z) - G_{sol}(Z\text{-}\mathbf{8}\text{-H}^+)}{2.303RT} = \frac{\Delta G_{sol}(E\text{-}\mathbf{8}\text{-H}^+)}{2.303RT} - \frac{\Delta G_{sol}(Z\text{-}\mathbf{8}\text{-H}^+)}{2.303RT} \quad (4)$$

Table S8.3 Relative energies (in kcal/mol) of neutral and protonated forms of **8a-d** in DCM obtained at the M06-2X/6-31+G(d)/SMD level of theory and corresponding ΔpK_a calculated according to eq. (4) for T = 298.15 K.

	R	ΔG_{sol} (Z-**8**-H$^+$)	ΔG_{sol} (E-**8**-H$^+$)	ΔpK_a ($E-Z$)
**8a**	H	258.1	255.33	-2.05
**8b**	COOMe	254.5	253.37	-0.84
**8c**	Me	258.9	257.01	-1.36
**8d**	MeO	260.0	256.85	-2.27

S8.3 Electronic transitions

Vertical excitation energies (VEEs) for the 3 lower electronically excited singlet states of *Z*-**8**-H$^+$, *E*-**8**-H$^+$, *Z*-**8** and *E*-**8** were calculated using the Linear Response (LR)[29] as well as corrected Linear Response (cLR) approach. MO analysis of the electronic transitions was performed.

Table S8.4 Ordering of low-lying electronically excited singlet states for Z-**8(a-d)**-H$^+$ in the gas phase obtained with the M06-2X/6-311++G(2df,2p)//M06-2X/6-31+G(d), CC2 and ADC(2)/aug-cc-pVTZ//M06-2X/6-31+G(d) approaches. Involved MOs are indicated when their weight is higher than 0.2.

	***Z*-form**				***E*-form**			
**state**	VEE (eV) (nm)	*f*	MOs (|weight|)	Type	VEE (eV) (nm)	*f*	MOs (|weight|)	Type
**M06-2X**								
**1**	2.96 (419)	0.11	60→63 (0.25)<br>62→63 (0.64)	(π,π*)<br>(π,π*)	2.62 (474)	0.045	58→63 (0.41)<br>62→63 (0.54)	(n,π*)<br>(π,π*)
**2**	3.21 (386)	0.00	58→63 (0.66)	(n,π*)	3.00 (414)	0.018	58→63 (0.33)<br>60→63 (0.48)<br>62→63 (0.35)	(n,π*)<br>(π,π*)<br>(π,π*)
**3**	3.78 (327)	0.36	60→63 (0.53)<br>61→63 (0.24)<br>62→63 (0.34)	(π,π*)<br>(π,π*)<br>(π,π*)	3.42 (362)	0.022	61→63 (0.69)	(π,π*)
**CC2**								
**1**	2.72 (456)	0.143	60→63 (0.49)<br>62→63 (0.84)	(π,π*)<br>(π,π*)	2.47 (501)	0.077	57→63 (0.45)<br>60→63 (0.23)<br>62→63 (0.81)	(n,π*)<br>(π,π*)<br>(π,π*)
**2**	3.21 (386)	0.006	57→63 (0.90)	(n,π*)	2.82 (440)	0.008	60→63 (0.72)<br>57→63 (0.59)<br>61→63 (0.22)	(π,π*)<br>(n,π*)<br>(π,π*)
**3**	3.55 (349)	0.397	60→63 (0.68)<br>62→63 (0.43)<br>61→63 (0.40)<br>57→63 (0.32)	(π,π*)<br>(π,π*)<br>(π,π*)<br>(n,π*)	3.27 (379)	0.048	60→63 (0.94)<br>61→63 (0.23)	(π,π*)<br>(π,π*)
**ADC(2)**								
**1**	2.64 (470)	0.118	60→63 (0.46)<br>62→63 (0.84)	(π,π*)<br>(π,π*)	2.33 (532)	0.047	62→63 (0.78)<br>57→63 (0.52)	(π,π*)<br>(n,π*)
**2**	3.01 (411)	0.001	57→63 (0.90)	(n,π*)	2.68 (462)	0.13	60→63 (0.72)<br>57→63 (0.54)<br>62→63 (0.26)<br>61→63 (0.23)	(π,π*)<br>(n,π*)<br>(π,π*)<br>(π,π*)
**3**	3.46 (359)	0.316	60→63 (0.73)<br>62→63 (0.42)<br>61→63 (0.35)	(π,π*)<br>(π,π*)<br>(π,π*)	3.14 (394)	0.033	61→63 (0.94)<br>60→63 (0.22)	(π,π*)<br>(π,π*)

Table S8.5 Theoretical characterization (orbital character, oscillator strength (*f*), and VEE (in eV) with corresponding λ_{max}^{theor} (in nm) in parentheses) of the $S_0 \rightarrow S_1$ transitions in the *Z* and *E* forms of compounds **8**-H^+ in DCM obtained at the cLR-TD-M06-2X/6-311++G(2df,2p)/SMD//M06-2X/6-31+G(d)/SMD level of theory. Theoretical absorption maxima and their differences are compared with experimental λ_{max}^{exp} values.

	Z				***E***				***E* - *Z***	
**ITI-H$^+$**	**Character ππ* / nπ***	***f***	***VEE*** **(λ_{max})** theory	exp	**Character ππ* / nπ***	***f***	***VEE*** **(λ_{max})** theory	exp	**Δ*VEE*** **(Δλ_{max})** theory	exp
**8a**	0.87 / -	0.16	2.96 (419)	2.62 (473)	0.58 / 0.36	0.06	2.75 (451)	2.45 (506)	-0.21 (32)	-0.17 (33)
**8b**	0.67 / -	0.08	2.93 (423)	2.74 (453)	0.61 / 0.52	0.00	2.88 (430)	2.56 (485)	-0.05 (7)	-0.18 (32)
**8c**	0.90 / -	0.26	2.94 (421)	2.58 (481)	0.60 / 0.34	0.10	2.65 (468)	2.42 (513)	-0.30 (47)	-0.16 (32)
**8d**	0.91 / -	0.46	2.86 (434)	2.38 (520)	0.62 / 0.26	0.23	2.48 (500)	2.21 (561)	-0.38 (66)	-0.17 (41)

Table S8.6 Theoretical characterization (orbital character, oscillator strength (*f*), and λ_{max}^{theor} (in nm) estimated as VEE) of the lowest allowed transitions in the *Z* forms of compounds **8** (transition $S_0 \rightarrow S_2$) and **8**-H^+ (transition $S_0 \rightarrow S_1$), obtained at the cLR-TD-M06-2X/6-311++G(2df,2p)/SMD(DCM)//M06-2X/6-31+G(d)/SMD(DCM) level of theory. For *Z*-**8c** and *Z*-**8d**, the data for the $S_0 \rightarrow S_1$ transition are also reported in parentheses due to relatively large *f* values. Theoretical absorption maxima and their shifts are compared with experimental λ_{max}^{exp} values.

	*Z-**8***				*Z*-**8**-H^+				***Difference***	
**ITI-H**	**Character ππ* / nπ***	***f***	λ_{max}^{theor}	λ_{max}^{exp}	**Character ππ* / nπ***	***f***	λ_{max}^{theor}	λ_{max}^{exp}	$\Delta\lambda_{max}^{theor}$	$\Delta\lambda_{max}^{exp}$
**8a**	0.82 / 0.25	0.10	378	432	0.87 / -	0.16	419	473	41	41
**8b**	0.63 / 0.21	0.10	379	432	0.67 / -	0.08	423	453	44	21
**8c**	0.45 / 0.49	0.08	(404)		0.90 / -	0.26	421	481	(17)	
	0.79 / 0.33	0.11	380	447					41	54
**8d**	0.53 / 0.43	0.20	(408)		0.91 / -	0.46	434	520	(26)	
	0.80 / 0.42	0.13	381	444					53	76

Table S8.7 VEEs for the 3 lowest singlet ES of *Z* and *E* forms of **8-H$^+$(8a-d)** calculated using the LR and cLR approaches at the TD-M06-2X/6-311++G(2df,2p)/SMD(DCM)//M06-2X/6-31+G(d)/SMD(DCM) level of theory.

ITI-H		Transition	Type ππ* / nπ*	Involved orbitals[a]	f	LR VEE (nm)	cLR VEE (nm)
**8a**	**Z**	$S_0 \rightarrow S_1$	0.87 / -	62 → 63 (0.66) / 60 → 63 (0.21)	0.157	418	419
		$S_0 \rightarrow S_2$	- / 0.66	58 → 63 (0.66)	0.001	374	377
		$S_0 \rightarrow S_3$	1.01 / -	60 → 63 (0.60) / 62 → 63 (0.21) / 59 → 63 (0.20)	0.531	326	327
	**E**	$S_0 \rightarrow S_1$	0.58 / 0.36	62 → 63 (0.58) / 58 → 63 (0.36)	0.061	451	451
		$S_0 \rightarrow S_2$	0.77 / 0.38	60 → 63 (0.43) / 58 → 63 (0.38) / 62 → 63 (0.34)	0.012	405	407
		$S_0 \rightarrow S_3$	0.93 / -	61 → 63 (0.62) / 60 → 63 (0.31)	0.105	332	367
**8b**	**Z**	$S_0 \rightarrow S_1$	0.67 / -	77 → 78 (0.67)	0.084	421	423
		$S_0 \rightarrow S_2$	- / 0.66	71 → 78 (0.66)	0.103	376	379
		$S_0 \rightarrow S_3$	1.12 / -	76 → 78 (0.50) / 75 → 78 (0.34) / 74 → 78 (0.28)	0.725	322	321
	**E**	$S_0 \rightarrow S_1$	0.61 / 0.52	71 → 78 (0.52) / 75 → 78 (0.32) / 76 → 78 (0.29)	0.001	426	430
		$S_0 \rightarrow S_2$	0.69 / -	77 → 78 (0.69)	0.022	409	416
		$S_0 \rightarrow S_3$	1.17 / -	76 → 78 (0.51) / 75 → 78 (0.40) / 74 → 78 (0.26)	0.066	315	334
**8c**	**Z**	$S_0 \rightarrow S_1$	0.90 / -	66 → 67 (0.63) / 65 → 67 (0.27)	0.259	423	421
		$S_0 \rightarrow S_2$	- / 0.65	62 → 67 (0.65)	0.001	373	375

		$S_0 \rightarrow S_3$	0.88 / -	65 → 67 (0.59) / 66 → 67 (0.29)	0.468	342	344
	**E**	$S_0 \rightarrow S_1$	0.60 / 0.34	66 → 67 (0.60) / 62 → 67 (0.34)	0.100	467	468
		$S_0 \rightarrow S_2$	0.74 / 0.38	65 → 67 (0.53) / 62 → 67 (0.38) / 66 → 67 (0.21)	0.012	403	405
		$S_0 \rightarrow S_3$	1.01 / 0.34	65 → 67 (0.40) / 62 → 67 (0.34) / 64 → 67 (0.32) / 66 → 67 (0.29)	0.243	346	358
**8d**	**Z**	$S_0 \rightarrow S_1$	0.91 / -	70 → 71 (0.64) / 69 → 71 (0.27)	0.458	439	434
		$S_0 \rightarrow S_2$	0.25 / 0.62	66 → 71 (0.62) / 69 → 71 (0.25)	0.013	371	373
		$S_0 \rightarrow S_3$	0.85 / 0.24	69 → 71 (0.58) / 70 → 71 (0.27) / 66 → 71 (0.24)	0.293	361	360
	**E**	$S_0 \rightarrow S_1$	0.62 / 0.26	70 → 71 (0.62) / 66 → 71 (0.26)	0.225	499	500
		$S_0 \rightarrow S_2$	0.67 / 0.41	66 → 71 (0.41) / 69 → 71 (0.47) / 68 → 71 (0.20)	0.012	402	404
		$S_0 \rightarrow S_3$	0.77 / 0.32	69 → 71 (0.48) / 70 → 71 (0.29) / 66 → 71 (0.32)	0.223	374	378

[a] The unsigned contributions (> 0.2) are reported.

Table S8.8 VEEs for the 3 lowest singlet ES of ***Z*** and ***E*** forms of **8(a-d)** in DCM calculated using the LR and cLR approaches at the TD-M06-2X/6-311++G(2df,2p)/SMD(DCM)//M06-2X/6-31+G(d)/SMD level of theory.

ITI		Transition	Type ππ* / nπ*	Involved orbitals[a]	f	LR VEE (nm)	cLR VEE (nm)
**8a**	**Z**	$S_0 \rightarrow S_1$	0.58 / 0.52	58 → 63 (0.52) / 61 → 63 (0.23) / 62 → 63 (0.35)	0.040	400	401
		$S_0 \rightarrow S_2$	0.82 / 0.25	62 → 63 (0.59) / 58 → 63 (0.25) / 61 → 63 (0.23)	0.099	378	378
		$S_0 \rightarrow S_3$	0.80 / 0.22	61 → 63 (0.55) / 56 → 63 (0.25) / 58 → 63 (0.22)	0.046	337	340
	**E**	$S_0 \rightarrow S_1$	mixed[b]	58 → 63 (0.54) / 61 → 63 (0.28) / 62 → 63 (0.31)	0.022	499	507
		$S_0 \rightarrow S_2$	mixed	61 → 63 (0.59) / 62 → 63 (0.37)	0.031	383	388
		$S_0 \rightarrow S_3$	mixed	61 → 63 (0.22) / 56 → 63 (0.26) / 58 → 63 (0.47)	0.099	310	314
**8b**	**Z**	$S_0 \rightarrow S_1$	0.55 / 0.54	73 → 78 (0.54) / 76 → 78 (0.26) / 77 → 78 (0.29)	0.029	398	399
		$S_0 \rightarrow S_2$	0.63 / 0.21	77 → 78 (0.63) / 73 → 78 (0.21)	0.102	377	379
		$S_0 \rightarrow S_3$	0.81 / 0.22	76 → 78 (0.55) / 69 → 78 (0.26) / 73 → 78 (0.22)	0.057	334	339
	**E**	$S_0 \rightarrow S_1$	mixed	77 → 78 (0.60) / 73 → 78 (0.32)	0.000	482	491

		$S_0 \rightarrow S_2$	mixed	$76 \rightarrow 78$ (0.70)	0.026	377	383
		$S_0 \rightarrow S_3$	mixed	$73 \rightarrow 78$ (0.48) / $77 \rightarrow 78$ (0.27) / $69 \rightarrow 78$ (0.29) / $67 \rightarrow 78$ (0.20)	0.001	301	307
**8c**	**Z**	$S_0 \rightarrow S_1$	0.45 / 0.49	$62 \rightarrow 67$ (0.49) / $66 \rightarrow 67$ (0.45)	0.080	403	404
		$S_0 \rightarrow S_2$	0.79 / 0.33	$66 \rightarrow 67$ (0.49) / $62 \rightarrow 67$ (0.33) / $65 \rightarrow 67$ (0.30)	0.108	381	380
		$S_0 \rightarrow S_3$	0.57 / 0.25	$65 \rightarrow 67$ (0.57) / $60 \rightarrow 67$ (0.25)	0.047	342	344
	**E**	$S_0 \rightarrow S_1$	mixed	$66 \rightarrow 67$ (0.57) / $62 \rightarrow 67$ (0.32)	0.035	511	520
		$S_0 \rightarrow S_2$	mixed	$65 \rightarrow 67$ (0.62) / $66 \rightarrow 67$ (0.29)	0.035	383	388
		$S_0 \rightarrow S_3$	mixed	$62 \rightarrow 67$ (0.46) / $65 \rightarrow 67$ (0.23) / $60 \rightarrow 67$ (0.23) / $66 \rightarrow 67$ (0.22)	0.129	318	322
**8d**	**Z**	$S_0 \rightarrow S_1$	0.53 / 0.43	$70 \rightarrow 71$ (0.53) / $66 \rightarrow 71$ (0.43)	0.198	408	408
		$S_0 \rightarrow S_2$	0.80 / 0.42	$66 \rightarrow 71$ (0.42) / $69 \rightarrow 71$ (0.32) / $70 \rightarrow 71$ (0.38)	0.131	384	381
		$S_0 \rightarrow S_3$	0.58 / 0.27	$69 \rightarrow 71$ (0.58) / $64 \rightarrow 71$ (0.27)	0.070	343	343
	**E**	$S_0 \rightarrow S_1$	mixed	$70 \rightarrow 71$ (0.57) / $66 \rightarrow 71$ (0.26) / $67 \rightarrow 71$ (0.20)	0.080	527	533
		$S_0 \rightarrow S_2$	mixed	$69 \rightarrow 71$ (0.59) / $70 \rightarrow 71$ (0.28)	0.054	389	392

$S_0 \rightarrow S_3$	mixed	69 → 71 (0.32) / 70 → 71 (0.24) / 66 → 71 (0.29) / 67 → 71 (0.28) / 68 → 71 (0.24)	0.216	333	336

[a] The unsigned contributions (> 0.2) are reported.
[b] For E-forms, it is difficult to assign n/π character of the MOs due to larger structural distortion.

Comments on changes of intensity ratios in the electronic spectra of ITI-H

The intensity ratio of the two bands in the *Z*-**8(a-d)**-H^+ series is well reproduced by the calculations (Fig. 6) and can be attributed to a different ππ* or nπ* and charge transfer (CT) character of the two transitions. Whereas in **8a**-H^+, **8b**-H^+, and **8c**-H^+ both $S_0 \rightarrow S_1$ and $S_0 \rightarrow S_3$ transitions have predominantly a ππ* character (see Table S8.6) -with the latter having moreover charge-transfer (CT) nature (see EDD plots in Fig. 6) giving rise to a more intense short-wavelength band- the $S_0 \rightarrow S_3$ transition in **8d**-H^+ has partially nπ* character and does not appear to be a strong CT transition. The observed changes are more related to the presence of a strong electron-donating group (**8d**-H^+) rather than to geometric aspects: despite the similar dihedral angle θ_2 in **8c**-H^+ and **8d**-H^+ (cf. Table S8.2), their spectra are notably different (Fig. 6c,d; red curves). Also, the ratio of the two bands corresponding to $S_0 \rightarrow S_3$ and $S_0 \rightarrow S_1$ transitions for **8c**-H^+ (containing a weak donor) is still smaller compared to **8a**-H^+ and **8b**-H^+, both theoretically as well as experimentally (Fig. 6a-c; red curves).

In the case of *E*-**8(a-d)**-H^+, the intensity ratio of the two bands corresponding to $S_0 \rightarrow S_3$ and $S_0 \rightarrow S_1$ transitions decreases with increasing ED character of the substituent, which is also reproduced, though somewhat overestimated, by theory. The larger intensity of the $S_0 \rightarrow S_3$ transition in the case of the unsubstituted (**8a**-H^+) and EWG (**8b**-H^+) derivatives is attributed to its dominant ππ* nature with significant charge transfer character (Table S8.6, Figs. S8.5 and S8.7).

S8.4 MO analysis of electronic transitions for protonated forms (ITI-H)

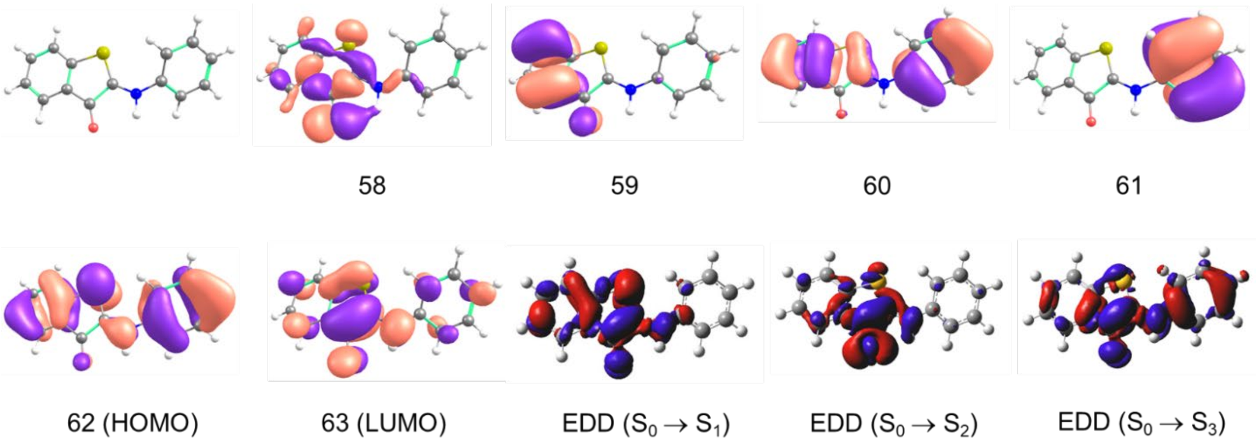

Figure S8.4 MOs (contour isovalue = 0.03) in electronic transitions from GS to low-lying ESs of *Z*-**8a**-H^+ in DCM and the corresponding electron density difference plots (EDDs, red = decrease, blue = increase, isovalue = 0.0015 au) obtained at the TD-M062X/6-311++G(2df,2p)/SMD//M06-2X/6-31+G(d)/SMD level of theory.

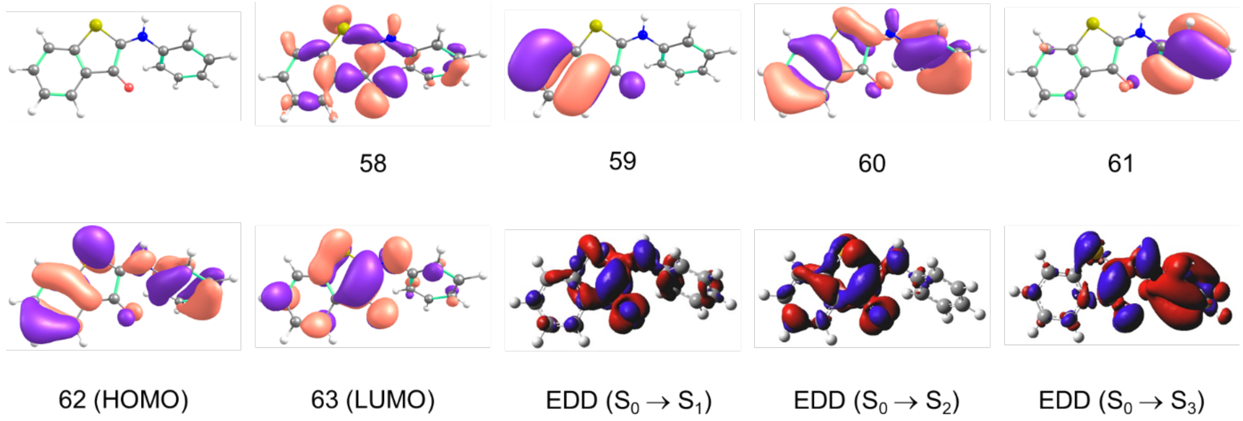

Figure S8.5 MOs (contour isovalue = 0.03) in electronic transitions from GS to low-lying ESs of *E*-**8a**-H^+ in DCM and the corresponding EDD plot (red = decrease, blue = increase, isovalue = 0.0015 au) obtained at the TD-M062X/6-311++G(2df,2p)/SMD//M06-2X/6-31+G(d)/SMD level of theory.

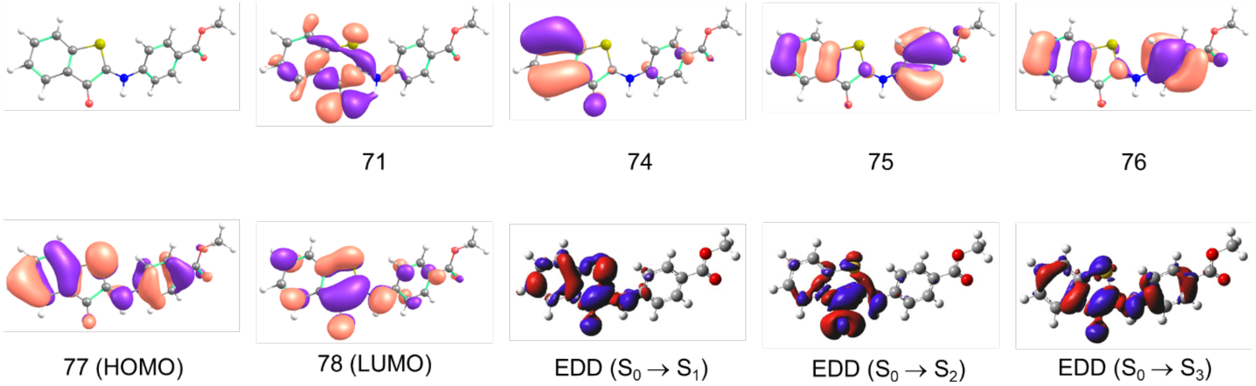

Figure S8.6 MOs (contour isovalue = 0.03) in electronic transitions from GS to low-lying ESs of *Z*-**8b**-H^+ in DCM and the corresponding electron density difference plot (EDD, red = decrease, blue = increase, isovalue = 0.0015 au) obtained at the TD-M062X/6-311++G(2df,2p)/SMD//M06-2X/6-31+G(d)/SMD level of theory.

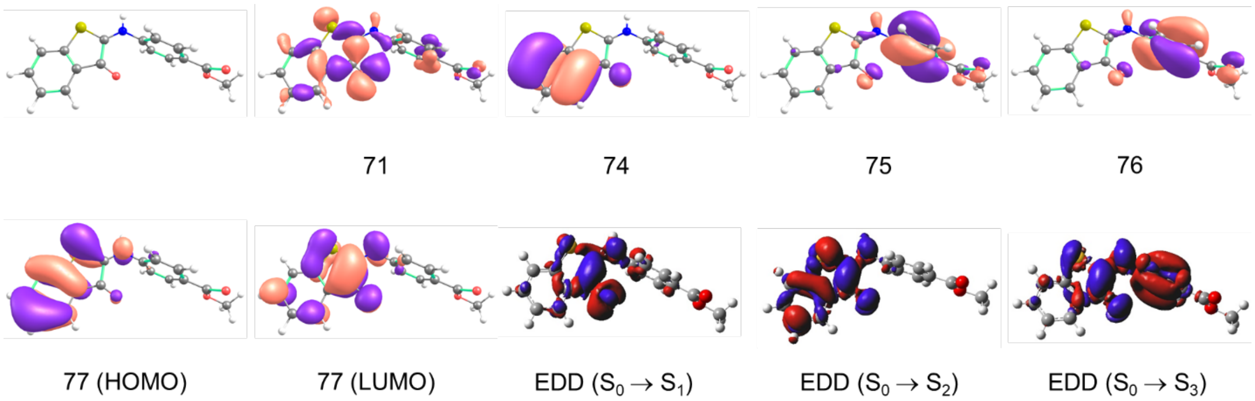

Figure S8.7 MOs (contour isovalue = 0.03) in electronic transitions from GS to low-lying ESs of *E*-**8b**-H^+ in DCM and the corresponding electron density difference plot (EDD, red = decrease, blue = increase, isovalue = 0.0015 au) obtained at the TD-M062X/6-311++G(2df,2p)/SMD//M06-2X/6-31+G(d)/SMD level of theory.

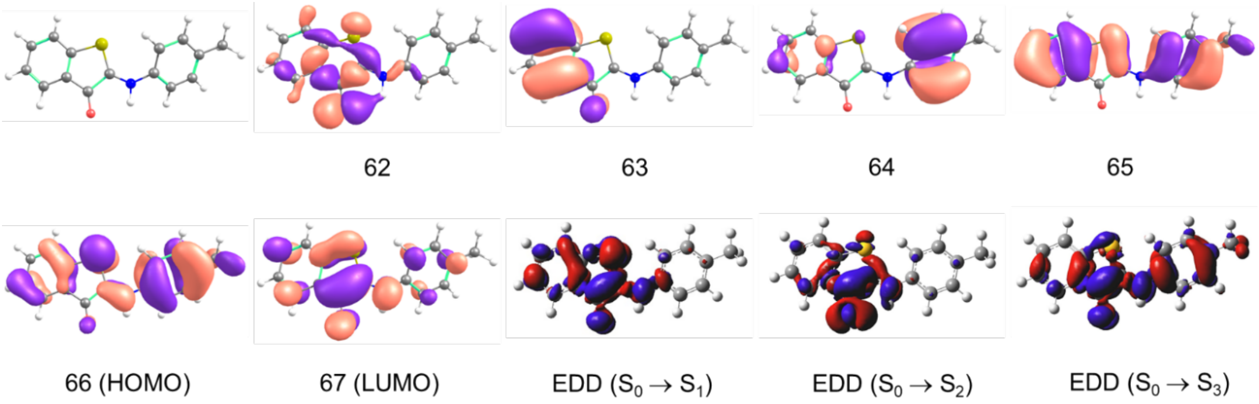

Figure S8.8 MOs (contour isovalue = 0.03) in electronic transitions from GS to low-lying ESs of *Z*-**8c**-H^+ in DCM and the corresponding electron density difference plot (EDD, red = decrease, blue = increase, isovalue = 0.0015 au) obtained at the TD-M062X/6-311++G(2df,2p)/SMD//M06-2X/6-31+G(d)/SMD level of theory.

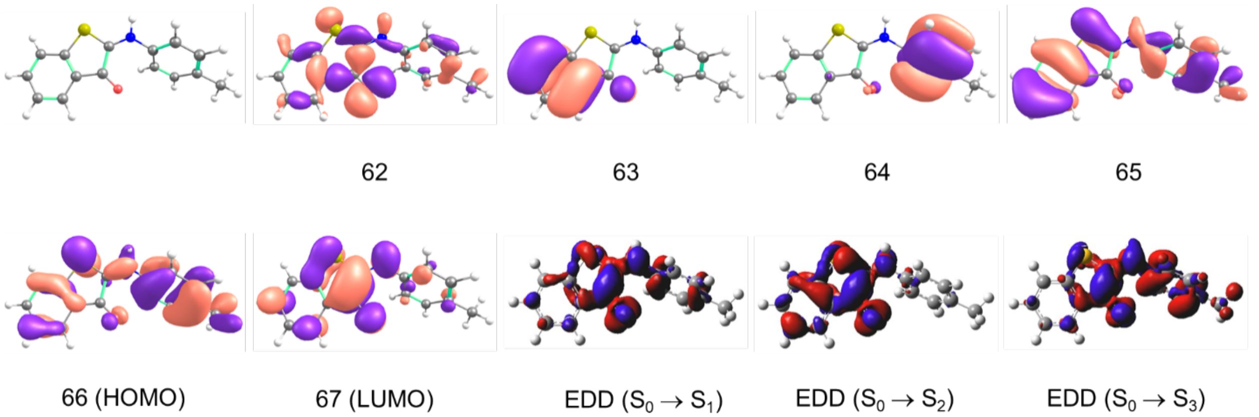

Figure S8.9 MOs (contour isovalue = 0.03) in electronic transitions from GS to low-lying ESs of *E*-**8c**-H^+ in DCM and the corresponding electron density difference plot (EDD, red = decrease, blue = increase, isovalue = 0.0015 au) obtained at the TD-M062X/6-311++G(2df,2p)/SMD//M06-2X/6-31+G(d)/SMD level of theory.

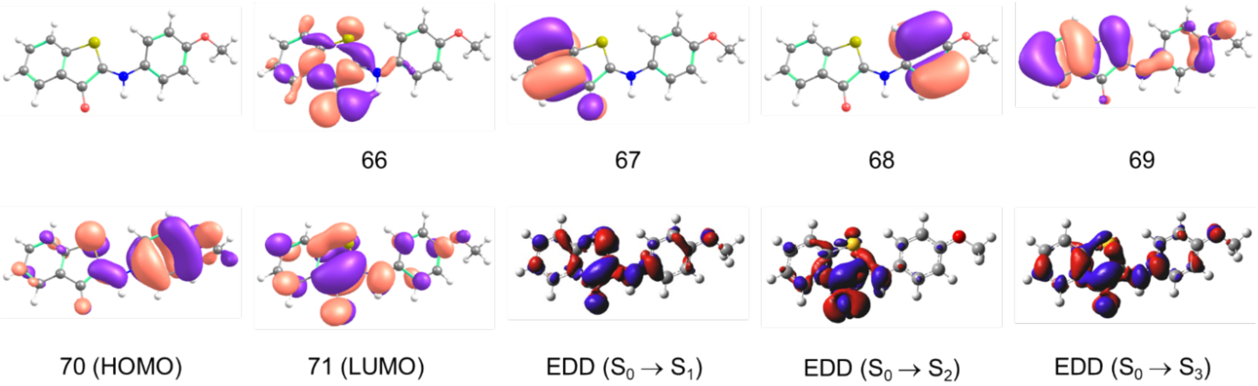

Figure S8.10 MOs (contour isovalue = 0.03) in electronic transitions from GS to low-lying ESs of *Z*-**8d**-H$^+$ in DCM and the corresponding electron density difference plot (EDD, red = decrease, blue = increase, isovalue = 0.0015 au) obtained at the TD-M062X/6-311++G(2df,2p)/SMD//M06-2X/6-31+G(d)/SMD level of theory.

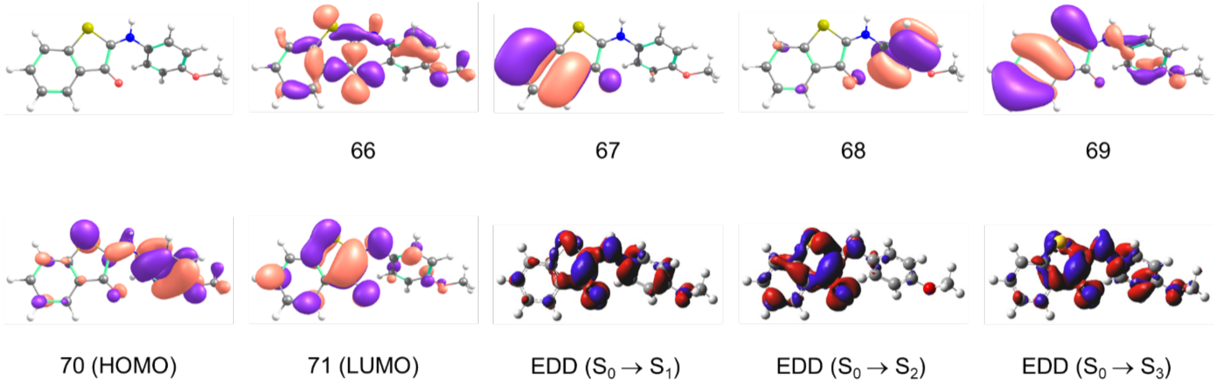

Figure S8.11 MOs (contour isovalue = 0.03) in electronic transitions from GS to low-lying ESs of *E*-**8d**-H$^+$ in DCM and the corresponding electron density difference plot (EDD, red = decrease, blue = increase, isovalue = 0.0015 au) obtained at the TD-M062X/6-311++G(2df,2p)/SMD//M06-2X/6-31+G(d)/SMD level of theory.

S8.5 MO analysis for neutral forms (ITI)

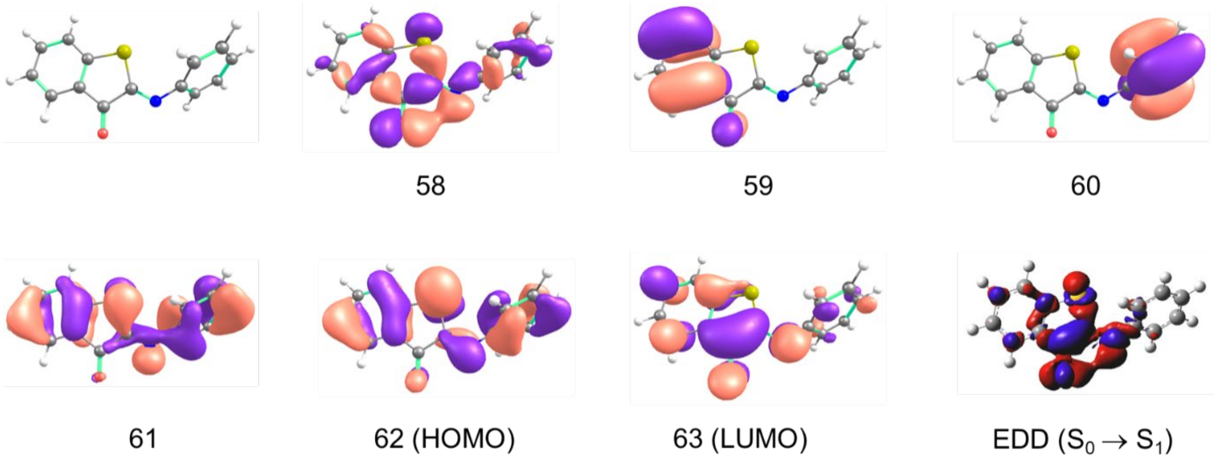

Figure S8.12 MOs (contour isovalue = 0.03) in electronic transitions from GS to low-lying ESs of *Z*-**8a** in DCM and the corresponding electron density difference plot (EDD, red = decrease, blue = increase, isovalue = 0.0015 au) obtained at the TD-M062X/6-311++G(2df,2p)/SMD//M06-2X/6-31+G(d)/SMD level of theory.

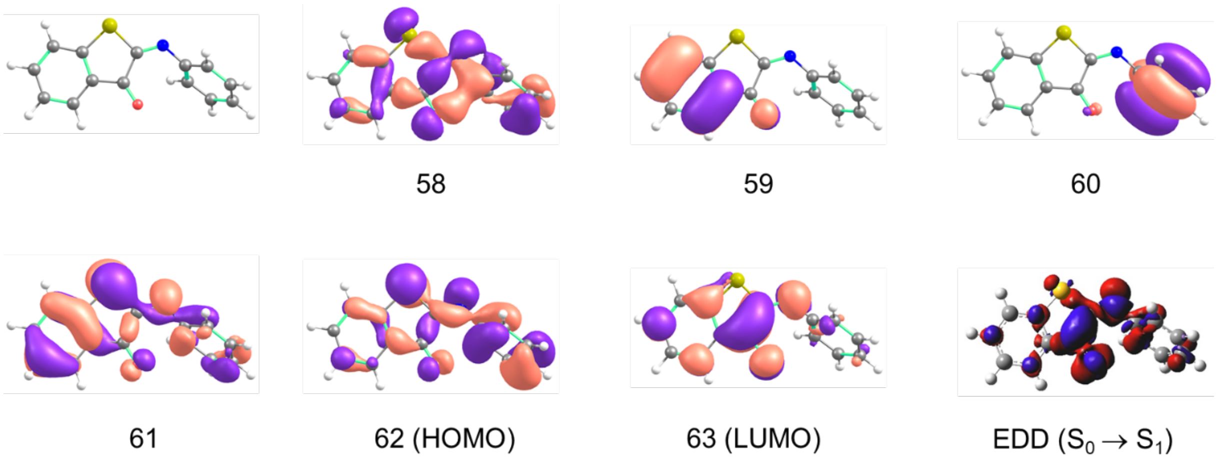

Figure S8.13 MOs (contour isovalue = 0.03) in electronic transitions from GS to low-lying ESs of *E*-**8a** in DCM and the corresponding electron density difference plot (EDD, red = decrease, blue = increase, isovalue = 0.0015 au) obtained at the TD-M062X/6-311++G(2df,2p)/SMD//M06-2X/6-31+G(d)/SMD level of theory.

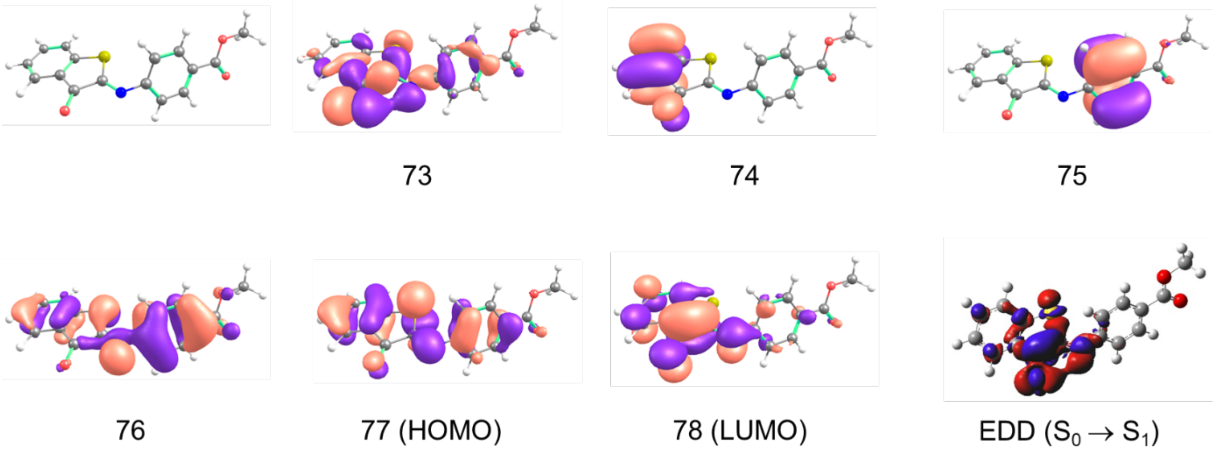

Figure S8.14 MOs (contour isovalue = 0.03) in electronic transitions from GS to low-lying ESs of *Z*-**8b** in DCM and the corresponding electron density difference plot (EDD, red = decrease, blue = increase, isovalue = 0.0015 au) obtained at the TD-M062X/6-311++G(2df,2p)/SMD//M06-2X/6-31+G(d)/SMD level of theory.

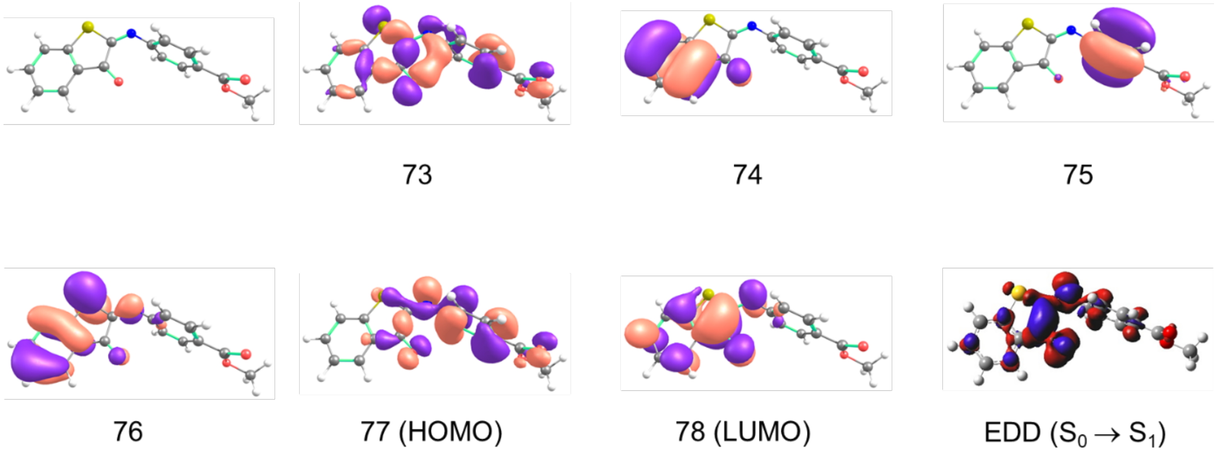

Figure S8.15 MOs (contour isovalue = 0.03) in electronic transitions from GS to low-lying ESs of *E*-**8b** in DCM and the corresponding electron density difference plot (EDD, red = decrease, blue = increase, isovalue = 0.0015 au) obtained at the TD-M062X/6-311++G(2df,2p)/SMD//M06-2X/6-31+G(d)/SMD level of theory.

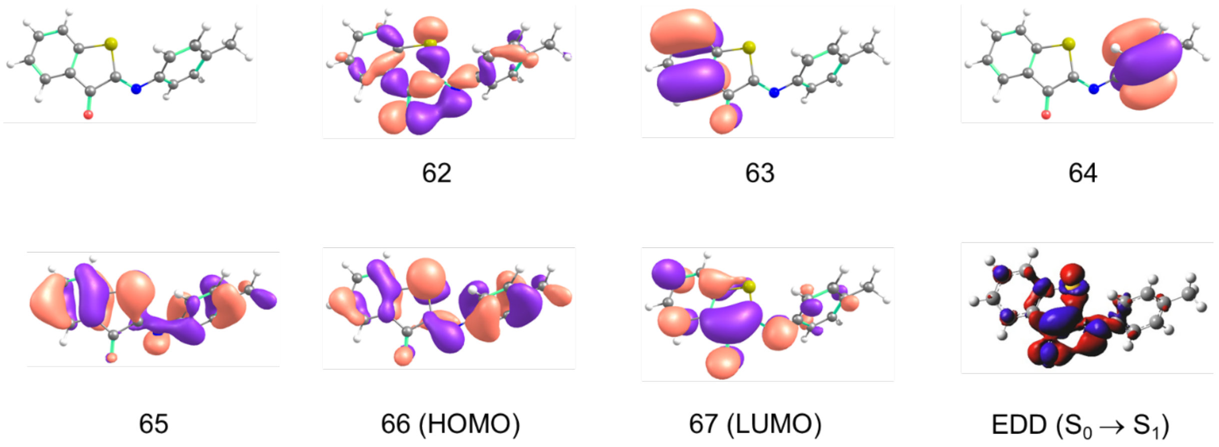

Figure S8.16 MOs (contour isovalue = 0.03) in electronic transitions from GS to low-lying ESs of *Z*-**8c** in DCM and the corresponding electron density difference plot (EDD, red = decrease, blue = increase, isovalue = 0.0015 au) obtained at the TD-M062X/6-311++G(2df,2p)/SMD//M06-2X/6-31+G(d)/SMD level of theory.

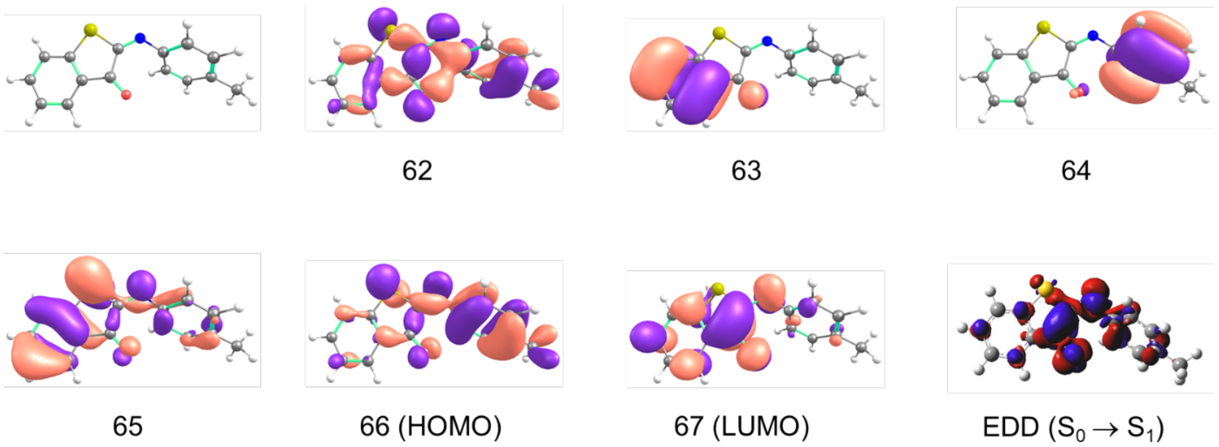

Figure S8.17 MOs (contour isovalue = 0.03) in electronic transitions from GS to low-lying ESs of *E*-**8c** in DCM and the corresponding electron density difference plot (EDD, red = decrease, blue = increase, isovalue = 0.0015 au) obtained at the TD-M062X/6-311++G(2df,2p)/SMD//M06-2X/6-31+G(d)/SMD level of theory.

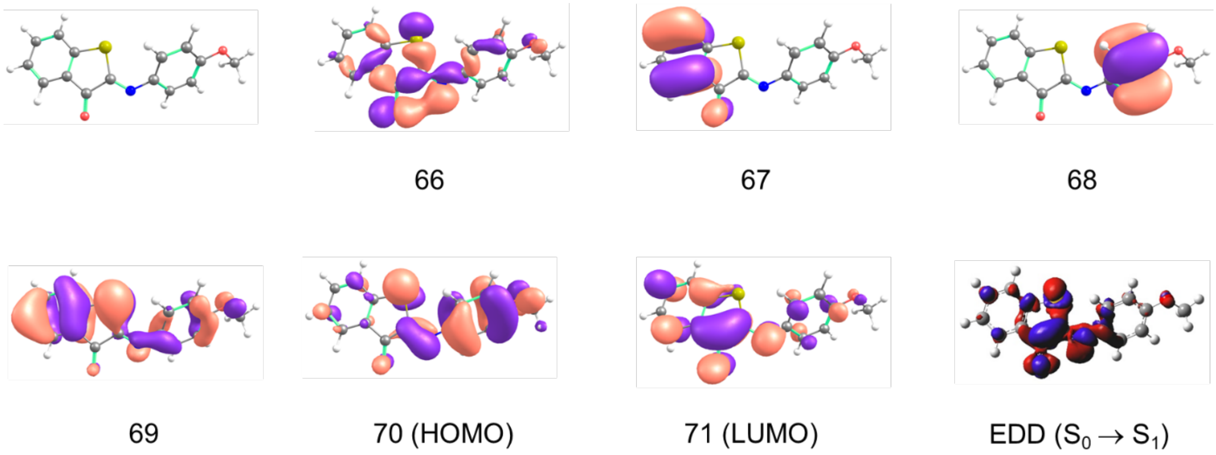

Figure S8.18 MOs (contour isovalue = 0.03) in electronic transitions from GS to low-lying ESs of *Z*-**8d** in DCM and the corresponding electron density difference plot (EDD, red = decrease, blue = increase, isovalue = 0.0015 au) obtained at the TD-M062X/6-311++G(2df,2p)/SMD//M06-2X/6-31+G(d)/SMD level of theory.

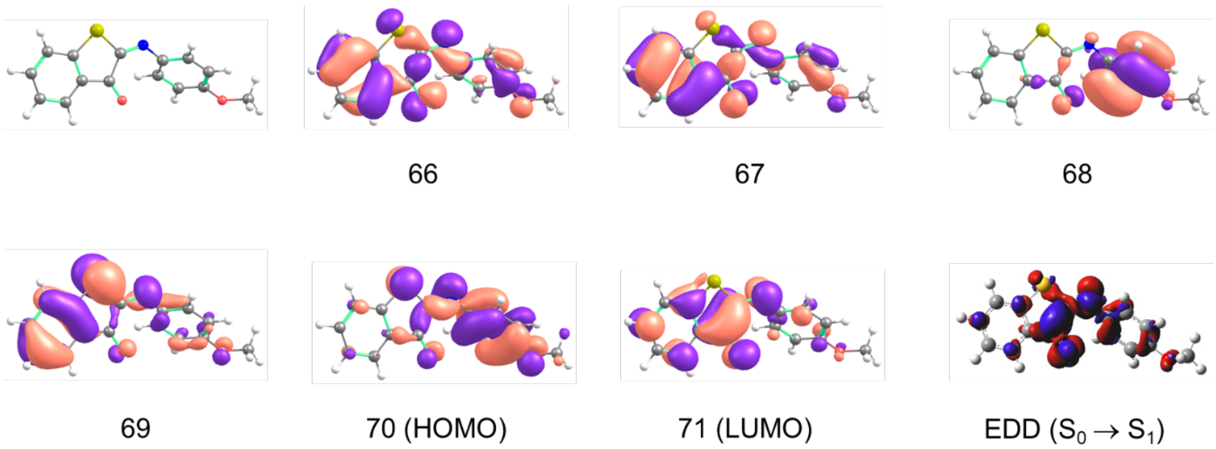

Figure S8.19 MOs (contour isovalue = 0.03) in electronic transitions from GS to low-lying ESs of *E*-**8d** in DCM and the corresponding electron density difference plot (EDD, red = decrease, blue = increase, isovalue = 0.0015 au) obtained at the TD-M062X/6-311++G(2df,2p)/SMD//M06-2X/6-31+G(d)/SMD level of theory.

S8.6 Excited state structure of Z-H$^+$ and ESIPT

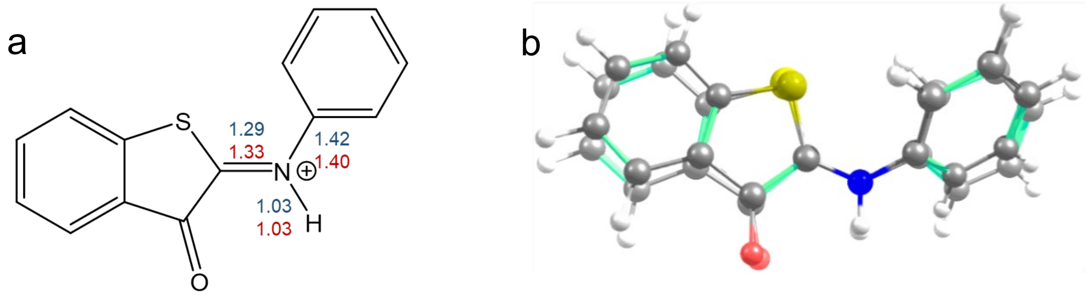

Figure S8.20 (a) Comparison of GS and ES (S_1) structures of *Z*-**8a**-H$^+$ (bond lengths are in Å, GS – blue, ES – red) and (b) superimposed GS and ES structures optimized at the (TD)-M06-2X/6-31+G(d)/SMD(DCM) level. *Comment: Upon excitation, Z-*8a*-H$^+$ gets slightly more open, but generally the molecular shape is preserved. The C=N bond length increases as a result of the HOMO-LUMO (π,π*) transition (LUMO has a nodal plane crossing the C=N bond, see Fig. S8.3).*

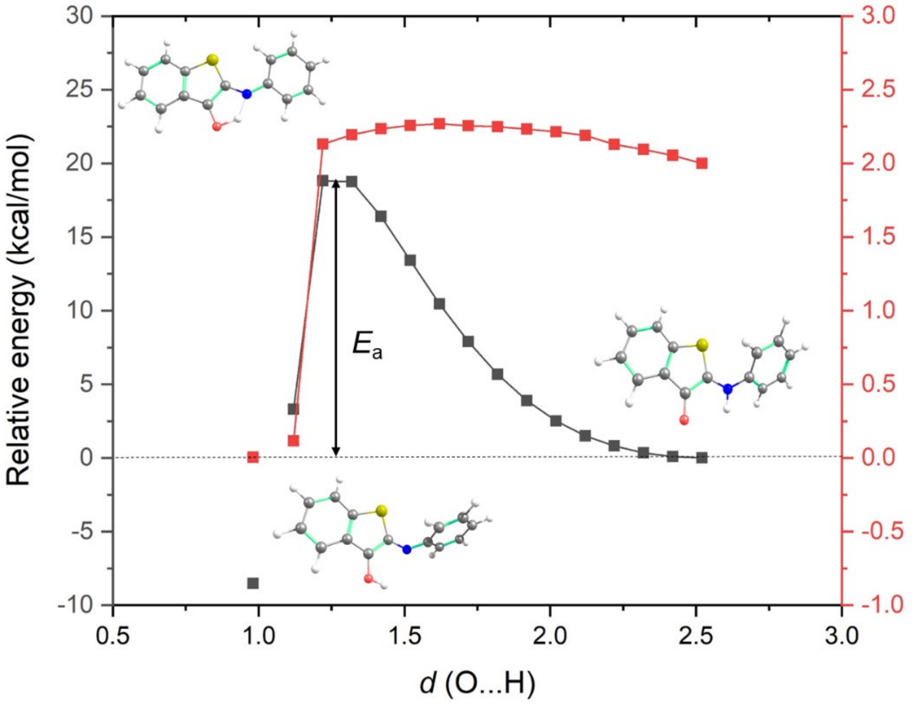

Figure S8.21 Dependence of the relative energy (in kcal/mol, black points) of the S_1 structures of *Z*-**8**-H$^+$ (with respect to optimized S_1 structure) on the O···H bond distance (in Å) for the ESIPT process (relaxed scan) obtained at the TD-M06-2X/6-31+G(d)/SMD(DCM) level of theory. The red points show the VEE energy values (in eV) for the $S_0 \rightarrow S_1$ transition obtained by the TD-DFT approach. *Note: The ESIPT structure is not a minimum. For the distances smaller than 1 Å, the structure relaxes towards the CI region, and thus the LR-TD-DFT approach is no more appropriate.*

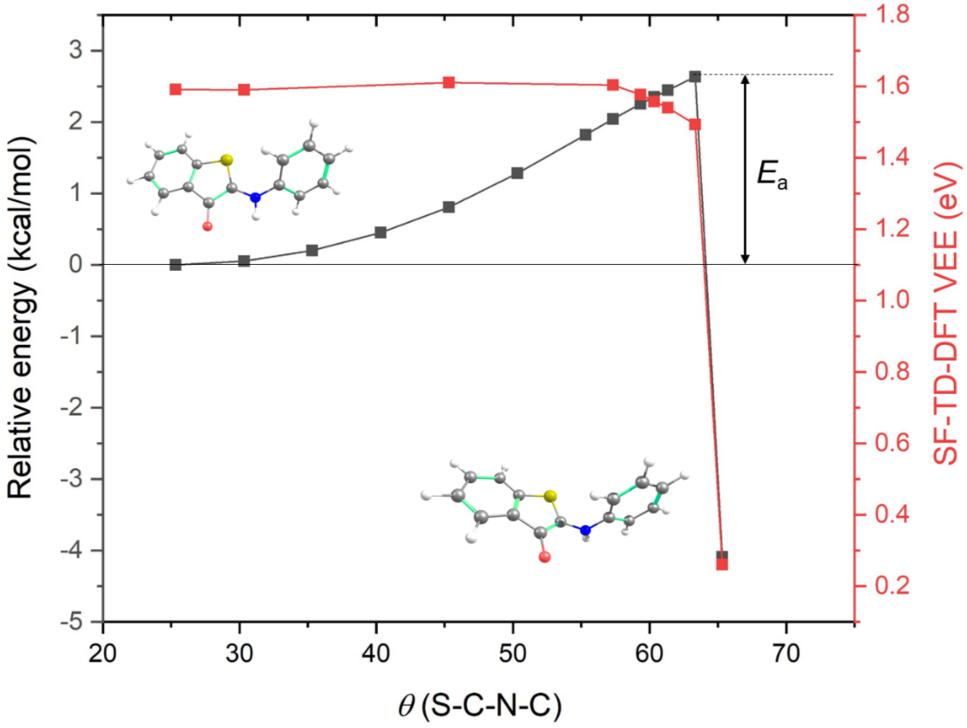

Figure S8.22 Dependence of the relative energy (in kcal/mol, black points) of the S_1 structures of *Z*-**8**-H^+ (with respect to optimized S_1 structure) on the torsion angle θ(S-C-N-C) obtained at the TD-M06-2X/6-31+G(d)/SMD(DCM) level of theory (relaxed scan). For the angles larger than 60°, the structure relaxes towards the CI region, and thus the LR-TD-DFT approach is no more appropriate. The red points show the VEE energy values (in eV) for the $S_0 \rightarrow S_1$ transition obtained by the SF-TD-DFT approach.

S8.7 Back-isomerization of protonated forms (Z-H$^+$ to E-H$^+$)

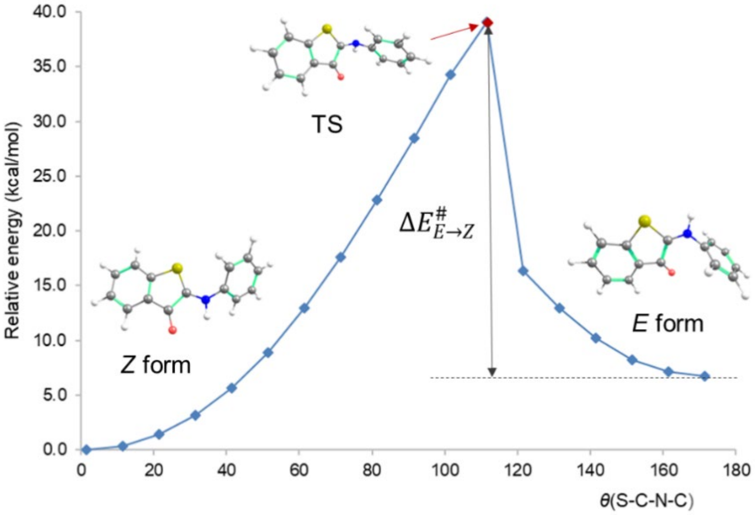

Figure S8.23 Dependence of the relative energy of the GS structures of *Z*-**8a**-H$^+$ in DCM on the torsion angle θ(S-C-N-C) obtained at the M06-2X/6-31+G(d)/SMD level of theory (relaxed scan). The red point corresponds to a true first-order saddle point (single imaginary frequency). *Comment: The application of the SF-TD-DFT approach revealed that the energy difference between the S_1 and S_0 PESs at the TS geometry of **8a**-H$^+$ in DCM is 0.76 eV, i.e., the two states are sufficiently separated to consider the GS DFT approach appropriate for determination of the back-isomerization barrier.*

S8.8 Back-isomerization of neutral forms (E to Z)

As reported in the previous study,[1] the TS structure corresponds to the nitrogen inversion (Fig. S8.2), and the nature of substituents dramatically affects the geometry of the TS. Whereas unsubstituted ITI (**8a**) and EWG derivative (**8b**) are twisted, EDG derivatives (**8c** and **8d**) are planar.

The application of the SF-TD-DFT approach revealed that the energy difference between the S_1 and S_0 PESs at the TS geometry of **8a** in DCM is 1.81 eV, i.e., the two states are again sufficiently separated to consider the GS DFT approach calculations as appropriate for determination of the back-isomerization barrier.

S9. Bibliography

1 M. W. H. Hoorens, M. Medved', A. D. Laurent, M. Di Donato, S. Fanetti, L. Slappendel, M. Hilbers, B. L. Feringa, W. J. Buma and W. Szymanski, *Nat. Commun.*, 2019, **10**, 2390.

2 Y. Zhao and D. G. Truhlar, *Theor. Chem. Acc.*, 2008, **120**, 215–241.

3 R. Ditchfield, W. J. Hehre and J. A. Pople, *J. Chem. Phys.*, 1971, **54**, 724–728.

4 A. D. Laurent and D. Jacquemin, *Int. J. Quantum Chem.*, 2013, **113**, 2019–2039.

5 A. V. Marenich, C. J. Cramer and D. G. Truhlar, *J. Phys. Chem. B*, 2009, **113**, 6378–6396.

6 S. Grimme, *J. Chem. Phys.*, 2003, **118**, 9095–9102.

7 M. Feyereisen, G. Fitzgerald and A. Komornicki, *Chem. Phys. Lett.*, 1993, **208**, 359–363.

8 C. Riplinger, P. Pinski, U. Becker, E. F. Valeev and F. Neese, *J. Chem. Phys.*, 2016, **144**, 024109.

9 G. D. Purvis and R. J. Bartlett, *J. Chem. Phys.*, 1982, **76**, 1910–1918.

10 D. G. Liakos, M. Sparta, M. K. Kesharwani, J. M. L. Martin and F. Neese, *J. Chem. Theory Comput.*, 2015, **11**, 1525–1539.

11 T. H. Dunning, *J. Chem. Phys.*, 1989, **90**, 1007–1023.

12 M. W. Schmidt, K. K. Baldridge, J. A. Boatz, S. T. Elbert, M. S. Gordon, J. H. Jensen, S. Koseki, N. Matsunaga, K. A. Nguyen, S. Su, T. L. Windus, M. Dupuis and J. A. Montgomery, *J. Comput. Chem.*, 1993, **14**, 1347–1363.

13 A. D. Becke, *J. Chem. Phys.*, 1993, **98**, 1372–1377.

14 Y. Shao, M. Head-Gordon and A. I. Krylov, *J. Chem. Phys.*, 2003, **118**, 4807–4818.

15 M. Caricato, B. Mennucci, J. Tomasi, F. Ingrosso, R. Cammi, S. Corni and G. Scalmani, *J. Chem. Phys.*, 2006, **124**, 124520.

16 O. Christiansen, H. Koch and P. Jørgensen, *Chem. Phys. Lett.*, 1995, **243**, 409–418.

17 M. J. Frisch, G. W. Trucks, H. B. Schlegel, G. E. Scuseria, M. A. Robb, J. R. Cheeseman, G. Scalmani, V. Barone, B. Mennucci, G. A. Petersson, H. Nakatsuji, M. Caricato, X. Li, H. P. Hratchian, A. F. Izmaylov, J. Bloino, G. Zheng, J. L. Sonnenberg, M. Hada, M. Ehara, K. Toyota, R. Fukuda, J. Hasegawa, M. Ishida, T. Nakajima, Y. Honda, O. Kitao, H. Nakai, T. Vreven, J. A. Montgomery, Jr., J. E. Peralta, F. Ogliaro, M. Bearpark, J. J. Heyd, E. Brothers, K. N. Kudin, V. N. Staroverov, R. Kobayashi, J. Normand, K. Raghavachari, A. Rendell, J. C. Burant, S. S. Iyengar, J. Tomasi, M. Cossi, N. Rega, J. M. Millam, M. Klene, J. E. Knox, J. B. Cross, V. Bakken, C. Adamo, J. Jaramillo, R. Gomperts, R. E. Stratmann, O. Yazyev, A. J. Austin, R. Cammi, C. Pomelli, J. W. Ochterski, R. L. Martin, K. Morokuma, V. G. Zakrzewski, G. A. Voth, P. Salvador, J. J. Dannenberg, S. Dapprich, A. D. Daniels, Ö. Farkas, J. B. Foresman, J. V. Ortiz, J. Cioslowski and D. J. Fox, Gaussian 09 (Revision A.02), Gaussian Inc., Wallingford CT, 2009.

18 M. J. Frisch, G. W. Trucks, H. B. Schlegel, G. E. Scuseria, M. A. Robb, J. R. Cheeseman, G. Scalmani, V. Barone, G. A. Petersson, H. Nakatsuji, X. Li, M. Caricato, A. V. Marenich, J. Bloino, B. G. Janesko, R. Gomperts, B. Mennucci, H. P. Hratchian, J. V. Ortiz, A. F. Izmaylov, J. L.

Sonnenberg, D. Williams-Young, F. Ding, F. Lipparini, F. Egidi, J. Goings, B. Peng, A. Petrone, T. Henderson, D. Ranasinghe, V. G. Zakrzewski, J. Gao, N. Rega, G. Zheng, W. Liang, M. Hada, M. Ehara, K. Toyota, R. Fukuda, J. Hasegawa, M. Ishida, T. Nakajima, Y. Honda, O. Kitao, H. Nakai, T. Vreven, K. Throssell, J. A. Montgomery, Jr., J. E. Peralta, F. Ogliaro, M. J. Bearpark, J. J. Heyd, E. N. Brothers, K. N. Kudin, V. N. Staroverov, T. A. Keith, R. Kobayashi, J. Normand, K. Raghavachari, A. P. Rendell, J. C. Burant, S. S. Iyengar, J. Tomasi, M. Cossi, J. M. Millam, M. Klene, C. Adamo, R. Cammi, J. W. Ochterski, R. L. Martin, K. Morokuma, O. Farkas, J. B. Foresman and D. J. Fox, Gaussian 16 (Revision A.03), Gaussian Inc., Wallingford CT, 2016.

19 F. Neese, *WIREs Comput. Mol. Sci.*, 2012, **2**, 73–78.

20 F. Neese, *WIREs Comput. Mol. Sci.*, 2018, **8**, e1327.

21 TURBOMOLE V7.3 2018, a development of University of Karlsruhe andForschungszentrum Karlsruhe GmbH, 1989-2007,TURBOMOLE GmbH, since 2007; available from http://www.turbomole.com.

22 E. R. Henry, *Biophys. J.*, 1997, **72**, 652–673.

23 I. H. M. Van Stokkum, D. S. Larsen and R. van Grondelle, *Biochim. Biophys. Acta - Bioenerg.*, 2004, **1657**, 82–104.

24 J. J. Snellenburg, S. Laptenok, R. Seger, K. M. Mullen and I. H. M. van Stokkum, *J. Stat. Softw.*, 2012, **49**, 1–22.

25 S. D. Christian and T. L. Stevens, *J. Phys. Chem.*, 1972, **76**, 2039–2044.

26 N. Mardirossian and M. Head-Gordon, *Mol. Phys.*, 2017, **115**, 2315–2372.

27 J. Tomasi, B. Mennucci and R. Cammi, *Chem. Rev.* 2005, **105**, 2999-3094.

28 C. Hättig, A. Köhn and K. Hald, *J. Chem. Phys.*, 2002, **116**, 5401–5410.

29 M. E. Casida, Recent Advances In Density Functional Methods: (Part I), 1995, 155-192.
